# The missing Nobels: Mapping the landscape of prestigious scientific prizes

**DOI:** 10.1371/journal.pone.0361810

**Published:** 2026-11-13

**Authors:** Ruchir Agarwal, Deivis Angeli, Patrick Gaule

**Affiliations:** 1 Global Talent Fund, Coquitlam, British Columbia, Canada; 2 Harvard University, Cambridge, Massachusetts, United States of America; 3 University of Bristol, Bristol, United Kingdom; Universidade Federal do Tocantins, BRAZIL

## Abstract

Prestigious recognition prizes, like the Nobel Prizes, can shape scientists’ career decisions and how science is seen. Yet the landscape of such prizes is not well understood. We screen roughly 2,700 international scientific prizes and rank the 99 most prestigious using a novel prestige index. Recognition is unevenly distributed across fields: physics, life sciences, and mathematics are heavily recognized relative to field size, while computer science, engineering, psychology, and the social sciences are under-served. Only three of the 99 prizes target early-career scientists.

## Introduction

In 1973, two computer scientists, Vinton Cerf and Robert Kahn, set out to solve a problem: how could machines on different networks talk to one another? Their solution, a set of rules called TCP/IP, became the basis of the Internet. Today it connects nearly every device, lab, and library on Earth. Yet no Nobel honors it: the Nobel categories were fixed by Alfred Nobel’s 1895 will, long before the information age, and computing has no home among them [1,2]. The will fixes the set of categories but not their boundaries: “one part to the person who made the most important discovery or invention in the field of physics; one part to the person who made the most important chemical discovery or improvement; one part to the person who made the most important discovery within the domain of physiology or medicine [...]” [1]. The boundaries have some give: the 2024 physics prize honored John Hopfield and Geoffrey Hinton for foundational work on machine learning, but the award was made in physics, on the grounds that neural networks connect to statistical physics. To Cerf and Kahn, recognition instead came from prizes created to fill such gaps (e.g., the Draper Prize and the Turing Award), but only in the early 2000s, roughly three decades after the work. Their story illustrates how major contributions can go unrecognized by established prizes when they fall outside traditional fields, and how long it can take for new prizes to fill the gap.

This paper takes this “Missing Nobel” idea as its starting point. It offers a novel systematic mapping of the landscape of prestigious scientific prizes and uses that map to diagnose institutional gaps: which fields receive the widely legible recognition that prizes provide, which career stages are rewarded, and how those rewards are structured. To answer these questions, we screen roughly 2,700 international scientific prizes and then select and rank the 99 most prestigious.

Prestigious prizes can shape how science is seen, funded, and pursued. Prizes function as both incentives, altering effort and risk-taking, and signals, updating beliefs about quality for funders, institutions, and the public. After an award, topics associated with a scientific prize grow faster and prizewinners’ earlier publications also gain more citations [3–6]. Because prize-giving institutions face reputational consequences for poor choices, these signals can carry information beyond what bibliometric indicators alone reveal: Turing Award and Nobel Prize laureates are rarely among the top-1% most-cited researchers in their fields (as we verify in our data); what distinguishes prizewinners is not volume of output but unusually innovative combinations of ideas [7]. Scientists in fields or career stages that are underserved by such prizes may lack these incentives and signals. Without a systematic map of where recognition concentrates, we cannot assess whether the signals guiding scientists’ career decisions are well-calibrated to the current structure of science.

To identify the most prestigious prizes, we build on previous work [8–10]. We focus on *recognition* prizes (those given for ex-ante unspecified contributions, like the Nobels) in science and technology. We adopt a broad definition of a recognition “prize”: besides cash-valued awards, we include honors such as fellowships and invited lectureships. We then include a prize in our list if it meets at least one of four criteria: appearing on three or more authoritative lists compiled by governments and ranking organizations; receiving high average ratings in prior expert surveys [9,10]; having recent winners who are predominantly among the top 1% most-cited researchers in their fields; or averaging at least 40 daily Wikipedia page views. We apply these selection criteria to a list of roughly 2,700 prizes. To avoid country-specific biases, we limit our attention to prizes open to scientists worldwide. Our method identifies the 99 most prestigious international prizes in science and technology.

To rank prizes, we build a novel measure of prize prestige. We conceptualize prestige broadly, as esteem and visibility within the scientific community and beyond it. Historical studies of scientific awards treat these as intertwined, since excellence is recognized and performed publicly, as well as achieved [11–13]. Our five indicators cover those different dimensions, as well as prize money and age. We use expert survey ratings [9,10], which capture peer esteem; Wikipedia page views, which capture demand for prize information [3,14,15]; media news mentions (from the MediaCloud database), which capture the broad supply of prize information; monetary value, which proxies institutional investment; and prize longevity, which proxies track record. Under the assumption that prize prestige explains most of the variance in these indicators, we extract their first principal component and use it as our prestige measure. Consistent with this hypothesis, this first principal component loads positively on every indicator and explains 49% of the total variance. The subsequent components, by contrast, capture trade-offs such as old-but-modest versus young-but-lavish prizes. This yields a transparent, theory-based prestige measure that allows direct comparison across disciplines.

The resulting map of scientific recognition reveals three patterns. First, prestige is being remade in real time: half of the top 99 prizes were created after 1980 and one-third after 2000. Aggregate annual prize money has grown even faster, roughly tripling since 2000, so a long history is not required to reach the top, although doing so seems increasingly expensive.

Second, coverage across fields is uneven even after normalizing by field size. To measure how much recognition each field receives, we assign each recent prizewinner (2015–2024) to a field based on their full publication record. We then compare the number of recognitions per field against measures of field size. As our two main measures of field size, we use the number of research academics at the top 150 US universities and the annual flow of new US PhDs—these proxy for the distribution of scientific work and investment across fields, or the number of potential prizewinners. Physics, the life sciences, and mathematics are the most recognized fields under both denominators; computer science, engineering, and the social sciences (excluding economics) are consistently under-served. Using US federal science funding shares or worldwide publication counts to measure field size, we find similar unevenness.

This unevenness across fields arguably has consequences for scientific progress, as (i) prizes shape the incentive structure that scientists face and (ii) prizes make scientific contributions legible across disciplinary boundaries. Other rewards, such as salaries and grants, may compensate scientists in fields with few prizes, but they do not make the field’s contributions visible to outsiders. Nor do we find such compensation: fields with fewer prizes show neither more patent-cited research nor higher private-sector pay. For instance, prizes can shape what non-scientists see as the most important disciplines and findings, affecting the popularity of public funding decisions and young people’s aspirations. Prizes can also inform scientists about what findings and methods are relevant in adjacent disciplines, allowing new methods to diffuse into a discipline and leading to new breakthroughs [16]. Fields with few or no prizes do not produce these widely legible signals.

Third, the incentive design of recognition prizes is narrow and backward-looking. Only three of the top 99 awards target early-career researchers (defined as prizes imposing any temporal restriction on eligibility, such as an age limit or a focus on recent work). Early-career prizes are arguably more socially valuable than late-career awards because they can function simultaneously as pull incentives—motivating effort through the prospect of status [17–20]—and as push incentives, easing resource constraints at the career stage when scientists are most productive [21] yet most funding-constrained [22]. Prizes are also narrow in how many people they name: the 784 awards made in 2015–2024 recognized 1.9 individuals on average, and 29 of the 99 prizes never divided an award, although science is increasingly done in teams. Beyond who is recognized, prize structures are almost completely backward-looking: of the 91 prizes with a monetary component, only four impose any conditions on how the money is spent. The remainder are one-time lump-sum payments with no link to future research output, mentorship, or milestones toward practical use.

We also note that prize prestige may not track research merit, and provide evidence. We compare where laureates are based with where each field’s most-cited researchers are: prizes with a national awarding body recognize domestically based researchers 40% more often than that benchmark implies, although these national biases mostly offset in the aggregate. Regardless of exactly how biased these prizes are, the gaps we document are still real, and, given that prize funding remains a popular endeavor, we provide guidance on how recognition prizes could better serve science.

This paper builds on two strands of prior research. First, it relates to the literature on *inducement* prizes—rewards offered ex ante for solving a specified problem. From the eighteenth-century Longitude Prize to modern technology challenges, such contests have been analyzed as mechanisms to direct R&D effort toward socially valuable goals [23–25]. A central lesson of this literature is that design matters: effectiveness depends on problem definition, eligibility breadth, and the match between prize structure and contestants’ incentives [23,26–28]. Recognition prizes—awarded ex post for exceptional achievement—have received far less formal analysis, despite serving distinct and arguably broader functions (e.g., they signal quality to funders, employers, and the public; they can shape which fields attract talent). By systematically mapping and ranking recognition prizes, this paper applies the design perspective of the inducement-prize literature to the ex post domain.

Second, we build on previous work on the measurement and mapping of scientific prestige. Earlier studies relied primarily on expert surveys benchmarked against the Nobels [9,10]; Meho [8] subsequently combined survey rankings with objective criteria to propose prize-based metrics for university rankings. Existing rankings remain limited in several ways. Qualitative lists, such as the one prepared for the International Congress of Distinguished Awards (a professional association for prize-giving organizations that maintains a roster of awards meeting its standards for administrative excellence), are subjective and difficult to update or extend. Most university-ranking systems that use prizes treat them as binary indicators, without weighting by prestige; the only exception is MosIUR, which uses weights from prior surveys [9]. We extend this literature by consolidating multiple data sources into a comprehensive list of international recognition prizes and a continuous prestige index, and, most importantly, by treating the recognition system itself as the object of analysis.

## Materials and methods

We build on previous efforts to systematically identify prestigious academic prizes, particularly the methodology developed by Meho [8]. We apply our selection criteria to the roughly 2,700 prizes that appear in previous listing efforts [8–10], in Wikipedia’s lists of prizes, or in the list of prizes considered eligible for UK immigration, and identify the 99 most prestigious academic awards using four criteria.

### Identifying recognition prizes

To focus on recurring scientific prizes of a truly international character recognizing individuals, we exclude prizes that:

- Have strict citizenship or national origin requirements
- Have only been given to individuals from or working in a single country in the last 10 years (or last 20 if given every two years)
- Did not distribute any awards in the past 10 years
- Lack accurate information on winners for the past decade (e.g., because there is no webpage for a prize or because information is very incomplete)
- Primarily recognize contributions in philosophy, education, or non-science fields
- Do not have systematic prizewinner selection criteria (e.g., a single individual selects the prizewinners, without an ex-ante specified scoring rule)
- Tend to recognize institutions rather than individuals

These criteria exclude widely recognized honors such as the MacArthur Fellowship (only given to residents or citizens) or the John Bates Clark Medal (only given to US-based researchers) in favor of describing a general and international picture of the recognition landscape.

We then select prizes that meet at least one of four criteria. First, we consider seven authoritative lists of prestigious awards compiled by government agencies, university ranking organizations, and academic publications (S1 Table in S1 Appendix); prizes appearing in at least three of these lists meet this criterion (42 prizes). Second, we incorporate expert survey data from two surveys [9,10] that received responses from 391 prizewinners and 536 academic leaders and highly cited researchers, respectively; prizes qualify if they received an average importance rating of at least 0.5 relative to a Nobel Prize and appeared in at least one authoritative list (55 prizes). Third, we take every prize that appears in at least one of the seven lists or was rated in either survey (the candidate pools for the first two criteria) and include those whose recent winners are predominantly (more than 50%) among the top 1% most-cited researchers in their fields, as measured by the Clarivate Highly Cited Researchers list (53 prizes). This citation criterion ensures that we do not miss prizes that consistently recognize the leading contributors of a field even when the prize itself is less visible. Fourth, we collect data on Wikipedia page views from 2020 to 2025; prizes averaging at least 40 daily views (the top 5% among the 2,700 prizes screened) qualify (26 prizes). Averaging over six years smooths over both the attention peaks near announcements [29,30] and the heterogeneity in how much visibility each prizewinner attracts, which varies with how easily their discoveries are communicated, their personalities, and other attributes [13]. Views of pages covering several prizes are allocated in proportion to prize money, and when a prize is only discussed as a section of a larger page, by the share of the page devoted to it. S2 Table in S1 Appendix reports how many prizes meet each criterion and how many meet no other. The 99 prizes are awarded by organizations in 18 countries and by three international bodies (S3 Table in S1 Appendix): US organizations award 33 of them and 33% of the 2015–2024 recognitions, European organizations 45 and 43%, and organizations in Asia and the Middle East 16 and 18%. No prizes given in mainland China make our list.

### Prize data

For each prize meeting our inclusion criteria, we record the year it was first awarded, its periodicity, recipients per award, and the cash value. A language model assisted the extraction and every entry was checked by hand against official sources. Prize money includes research funds, if any; money per winner divides it by the number of recipients, and money per year by the award interval. We also code each prize’s awarding organization, its country, and the type of funding source (philanthropic, corporate, learned society, government, or university) from statutes, websites, annual reports, and press announcements (see the note to S11 Fig in S1 Appendix for details). For each prize we also list every recipient from 2015 to 2024 from the awarding body’s own records and match each recipient to an OpenAlex author profile. We code only the people a prize names as laureates, since they are the individuals directly recognized. Only four awards in 2015–2024 involved teams. The Breakthrough Prize in Fundamental Physics named the leaders of the supernova search teams (2015) and of the neutrino-oscillation experiments and the LIGO collaboration (2016), and those leaders are the laureates we count. Its 2020 award to the Event Horizon Telescope Collaboration and the Royal Society’s 2022 Copley Medal to the Oxford–AstraZeneca vaccine team went to the collaboration as a whole; each counts as one recognition event, assigned to the field of the scientist who accepted it. We revisit this issue in the Discussion.

### Ranking prizes

To rank the prizes selected by our inclusion criteria, we combine five prestige indicators: expert survey ratings [9,10], Wikipedia page views, media news mentions, prize age, and prize money. We extract a signal from these indicators using principal component analysis, the idea being that the major common factor behind those indicators is prize prestige, which is captured by the first principal component.

To construct the indicators, we take the logarithm of prize age, daily page views, media mentions, and prize money. For daily page views, media mentions, and prize money, we add one before taking the log to avoid infinities. Media news mentions are measured as the number of unique news articles mentioning each prize name in the Media Cloud Online News Archive [31] between January 2020 and December 2025, the same window used for Wikipedia page views. Media news mentions are counted based on Media Cloud’s national source collections (about 15,000 news sources in 100+ countries), queried using the strategy described in S4 Table in S1 Appendix. The pairwise correlations between our prestige indicators (S5 Table in S1 Appendix) show that media news mentions and Wikipedia page views are the most strongly correlated pair (*r* = 0.74), followed by expert survey ratings and page views (*r* = 0.68), while prize age and money per prize are negatively correlated (r=−0.47).

The first principal component of our normalized indicators explains 49% of total variance and loads positively on all five indicators, supporting a single-factor prestige interpretation; its weights are reported in the Results section. For the 23 prizes lacking survey ratings, we predict missing values using available indicators and field information. Specifically, we regress survey ratings on quartile indicators of page views, prize age, and money per award period, field fixed effects, and indicators for meeting inclusion criteria 1 and 3. We then apply a 50% discount to these predicted survey ratings, since omission from the surveys likely signals lower standing. The final prize ranking and tier system are derived by using the PCA weights to create a prestige index. We further classify prizes into three tiers. Summing recipients per year over all listed prizes gives the total number of recognition events they generate. After ranking, we assign a prize to Tier 1 if cumulative recognition events up to and including that prize do not exceed 10% of the total. Tier 2 contains the subsequent prizes whose cumulative share does not exceed 30%, and Tier 3 contains the remainder.

### Measuring prize density

To identify which fields are under- or over-supplied by top international prizes, we compare the supply of prizes in a field to two different proxies of field size. As our primary measure of field size, we use the number of research academics teaching in PhD programs at the top 150 US universities (according to the 2019 US News ranking), drawing on faculty data from Angeli and Lowe [32], who collected the universe of research academics in those programs and matched them to profiles of academics in OpenAlex—an open bibliographic dataset comparable with Elsevier’s Scopus or Clarivate’s Web of Science [33]. Of the roughly 100,000 academics found, 87,000 are matched to an OpenAlex profile. Then, for each academic, we identify the OpenAlex subfield containing most of their publications. As a secondary measure of field size, we use the number of new PhDs produced in the US in each field, extracted from the 2023 Survey of Earned Doctorates by the National Center for Science and Engineering Statistics. The data covers all research doctoral degrees, 99% of which are PhDs.

To calculate the number of prizes directed to each field, we list all prizewinners from 2015 to 2024 and also assign them to fields based on the subfield in which they have the most publications. We then group the 252 OpenAlex subfields into 26 fields that are readily interpretable and that harmonize with the PhD student data (the secondary measure of field size). For instance, we group subfields that share substantial institutional overlap (e.g., Physics and Astronomy) or that OpenAlex does not separate cleanly (e.g., the subfields in which political scientists and sociologists work cannot be easily separated, so we keep them together).

We measure prize density across fields by dividing the number of yearly recognition events associated with a field by that field’s size (each co-recipient of a shared prize counts as a separate recognition event, and a researcher who receives multiple prizes is counted once per prize). Because each recognition event is assigned to a field based on the individual winner’s publication record, inter- or multidisciplinary prizes are distributed across fields according to the specializations of their winners. As an additional robustness check, we divide observed yearly recognitions by US federal research funding, which may better capture national and public interest in each field (S1 Fig in S1 Appendix).

## Results

### Mapping and ranking prestigious prizes

Our methodology identifies the 99 most prestigious scientific prizes, ranked in S6 Table in S1 Appendix. For ranking the prizes, the PCA-based prestige indicator puts a weight of 26.3% on expert survey ratings, 29.8% on Wikipedia page views, 28.5% on media news mentions, 2.1% on prize age, and 13.2% on prize money. This first principal component explains 49% of total variance in our prestige indicators, nearly twice the share of the second component (27%), which loads almost exclusively on prize age (+0.75) and prize money (−0.64)—with near-zero loadings on survey ratings, page views, and news mentions—capturing an old-but-modest versus young-but-lavish trade-off.

These 99 prizes are on average 61 years old and have 78 daily Wikipedia page views (Table 1). They distribute on average USD 306,000 to each of their winners (median USD 167,000), with each prize distributing on average USD 650,000 per year. Together, they distribute about USD 64 million per year.

**Table 1:** Summary statistics.

Variable	Median	Mean	SD	Min	Max	N
Survey Rating	0.57	0.61	0.16	0.30	1.00	76
Daily Page Views	13.9	77.9	208.8	0.0	1,137.9	99
News Mentions	17	300	1,035	0	6,802	99
Prize Age	43	61	58	6	294	99
Period (Years)	1.0	1.4	0.8	1.0	6.0	99
Yearly Winners	1.50	1.63	1.43	0.25	12.00	99
Money per Year	187	650	1,573	0	9,000	99
Money per Prize	250	503	637	0	3,000	99
Money per Winner	167	306	456	0	3,000	99

*Note*: This table shows summary statistics for the 99 most prestigious recognition prizes. Survey Rating: expert ratings of prize importance relative to the Nobel [9,10]. Daily Page Views: average daily Wikipedia page views, 2020–2025. News Mentions: unique news articles mentioning the prize in the Media Cloud archive, 2020–2025. Prize Age: years since first awarded, as of 2025. Period: years between award rounds (1 = yearly). Yearly Winners: recipients per round divided by the period. Money per Year, per Prize and per Winner: prize money per round divided by the period, by the number of prizes per round, and by the number of recipients per round. Money in thousands of USD.

In terms of ranking, we assign eight prizes to the top tier, twelve to the second, and 79 to the third. The 99 prizes generate about 162 recognition events (one person recognized with one prize) per year. Prizes in computing and electrical engineering, mathematics, and chemistry tend to rank higher in our list, while prizes in other engineering fields tend to rank lower (S7 Table in S1 Appendix). Physics and astronomy has 14 prizes and an average rank close to the overall median; life and health sciences has the most prizes (25). Eighteen prizes are classified as multidisciplinary, with seven further prizes focused on environmental themes.

Our tier-based classification system is largely robust to different weighting schemes. We simulate 1,000 rankings with random weights, showing how the ranking of each prize would vary in S8 Table in S1 Appendix. Specifically, we draw weights from a Dirichlet(1,1,1,1,1) distribution, which is uniform over the simplex. Overall, 91% (61%) of prizes fall in the same tier as the one implied by our PCA-based ranking in over 50% (90%) of the simulations. At the top, for prizes like the Nobels and Turing Award, our PCA-based ranking sits tightly between the 5^th^ and 95^th^ percentiles of the random rankings, indicating little uncertainty about how they should be ranked. There is more variation in the simulated ranks of lower-ranked prizes, in good part due to the negative correlation between prize money and prize age.

### Explosion in recognition prizes

The number of established prestigious prizes rose sharply after 1960 (Fig 1), consistent with prior work documenting that the number of scientific prizes in general grew faster than the number of disciplines after 1980 [14]. The pace at which new highly prestigious prizes are created accelerates around the 1960s, showing that while having a long history might increase a prize’s prestige, it is clearly not a necessary requirement for making the top list. Accordingly, there is little correlation between expert ratings and prize age (Fig 2a).

**Fig 1:**
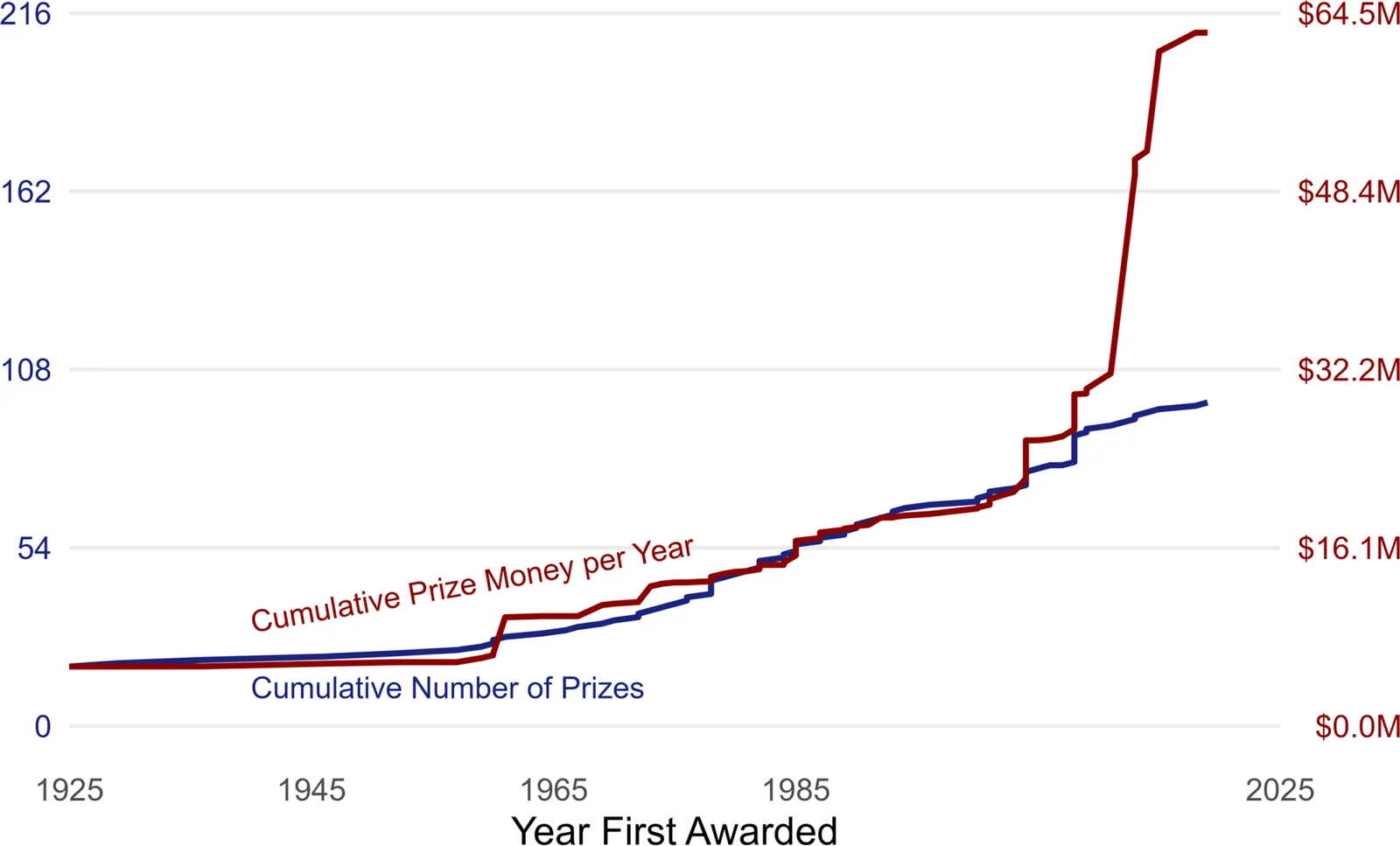
The explosion in prestigious recognition prizes. *Note*: The figure plots time series for the number of existing most-prestigious prizes each year (left axis) and the total yearly prize money given out by those prizes (right axis). The vertical axes are scaled so that the number of prizes and the prize money have the same base height in 1925. The series covers 98 prizes, excluding the discontinued Max Planck Research Award, the predecessor of the included Max Planck–Humboldt Research Award.

**Fig 2:**
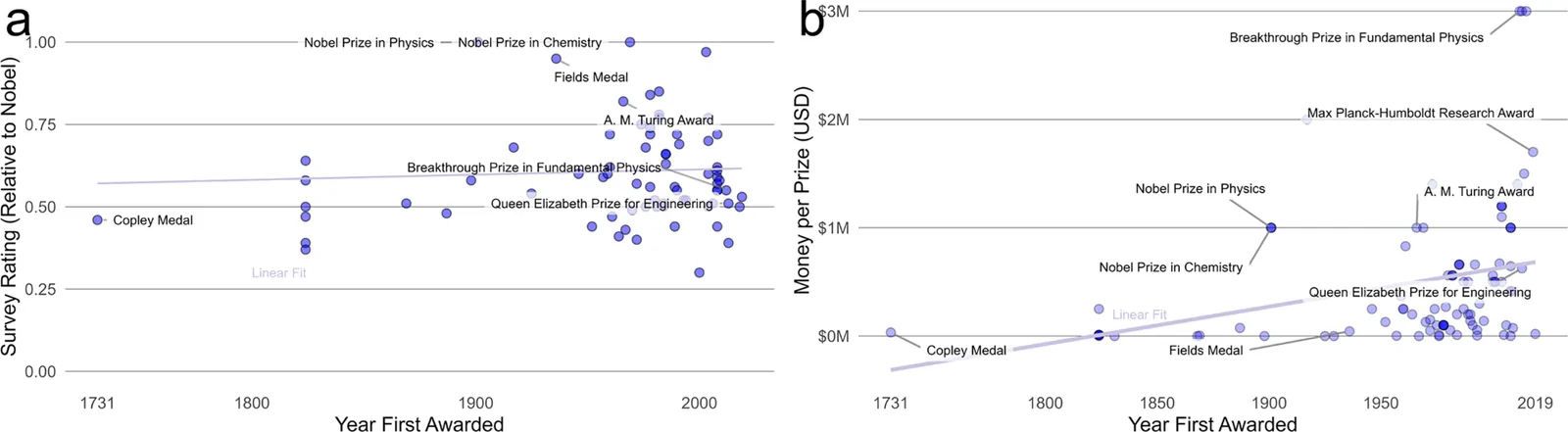
Prize age versus expert rating and prize money (a) Expert survey rating; (b) Prize money per award. *Note*: Both panels plot year first awarded on the horizontal axis. Panel (a) plots expert survey rating (normalized relative to the Nobel Prize) for the 75 prizes with observed survey ratings; ratings from prior expert surveys [9,10]. Panel (b) plots prize money per award (which may be shared among multiple winners) for 98 prizes. Both panels exclude the discontinued Max Planck Research Award, the predecessor of the included Max Planck–Humboldt Research Award.

The creation of new prestigious prizes has been driven by diverse actors. Governments established prizes like the Abel Prize (Norway, 2003) and the Queen Elizabeth Prize for Engineering (UK, 2013). Silicon Valley entrepreneurs funded the Breakthrough Prize family, the most visible recent example of a privately founded prize. International organizations like the ICTP and the International Mathematical Union collaborated to create the ICTP Ramanujan Prize for young mathematicians.

The money distributed by prestigious prizes increased even faster. Since 2000, the number of prestigious prizes has increased by about 40%, while the cash values distributed yearly have approximately tripled (Fig 1). Before 2000, growth in yearly prize money seems to more or less match the growth in the number of prizes, but that is probably an understatement since our figures only take into account a prize’s current cash value while cash prizes have likely increased faster than inflation (e.g., the inflation-adjusted value of a Nobel Prize in the early 1900s would be about USD 500,000 in 2025). Accordingly, we find a strong (negative) correlation between prize age and money distributed per prize awarded (Fig 2b).

Hence, while it has been possible to create new prestigious prizes, the *effective* cost to do so has increased, perhaps because new prizes have to compete for the public’s limited attention with more already-prestigious prizes than their predecessors did. While we do not provide causal evidence that larger purses lead to more prestige, we note that although there is only a modest raw correlation between prize money and online popularity (S2 Fig in S1 Appendix), prize money becomes a statistically significant predictor of page views after controlling for prize age, i.e., 1% more prize money (per award) is associated with 0.08% more page views (*p* = 0.04) and 0.22% more media news mentions (*p* < 0.001). These associations are correlational; reverse causality, whereby more prestigious prizes attract larger endowments, is also plausible.

### Gaps across fields

To identify which fields are over- or under-served, we divide the supply of yearly recognitions offered by the 99 top prizes by field size, the latter proxied by the number of researchers in top institutions or PhD students in each field. Several fields are consistently among the most recognized under both denominators. Physics & Astronomy and Microbiology & Immunology show the highest award density per academic, each receiving about five elite recognitions per thousand top researchers (Fig 3). Neuroscience, Mathematics, and Molecular Biology & Genetics also rank in the top seven under both measures (Fig 4). The earth and environmental sciences (Climate & Ocean Sciences, Geosciences, Ecology & Evolution) form a second cluster of well-recognized fields.

**Fig 3:**
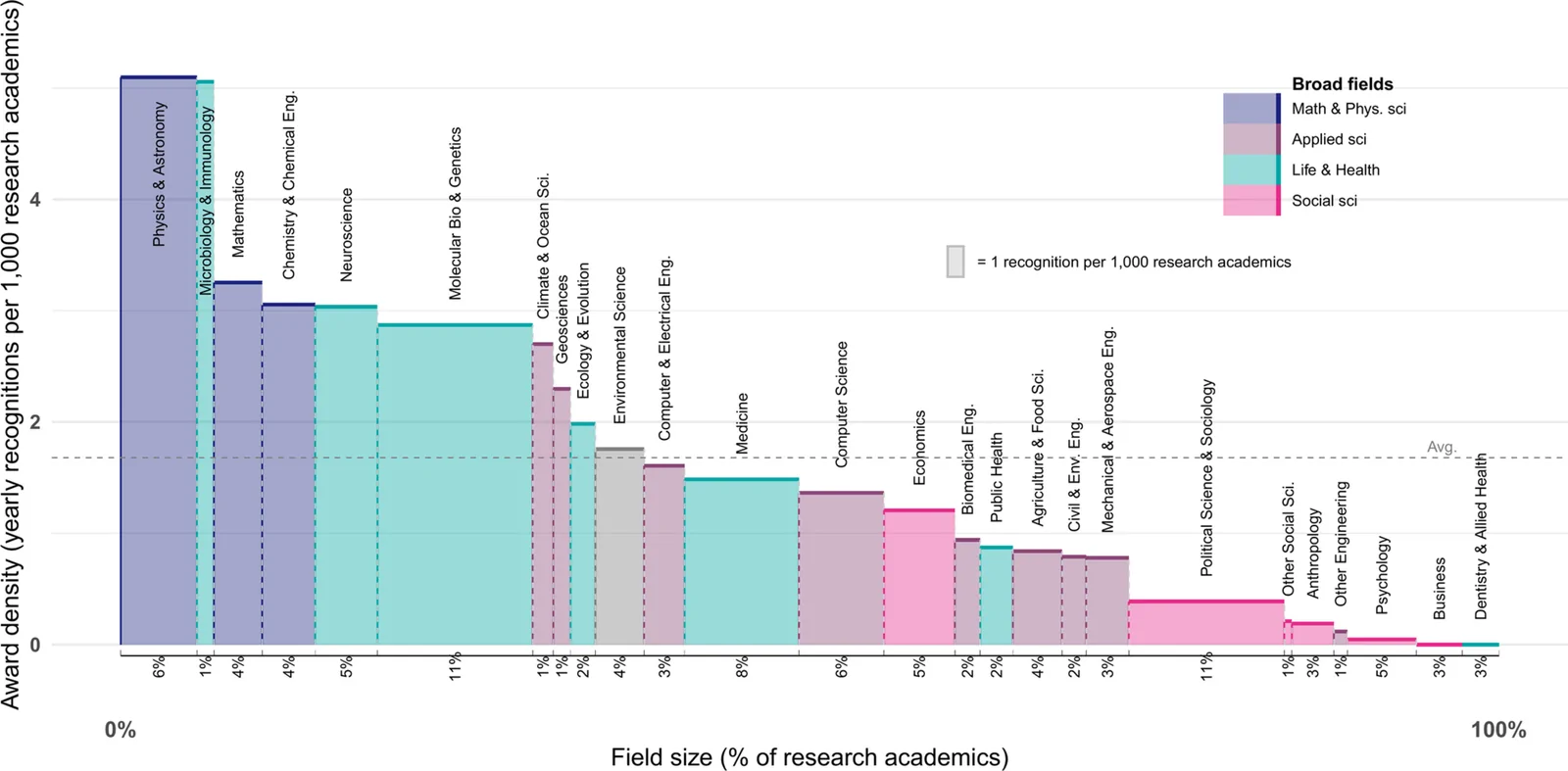
Award density by field (research academics denominator). *Note*: The figure plots the density of recognition events created by the 99 most prestigious prizes across 26 field groups. Each recognition event is assigned to a field based on the winner’s primary research field (openalex.org author profile). Recognition events from 2015–2024 are averaged over 10 years. Field size is measured based on the number of research academics at the top 150 US universities. Density regions are colored by broad fields. The relative size of each field is displayed under each bar.

**Fig 4:**
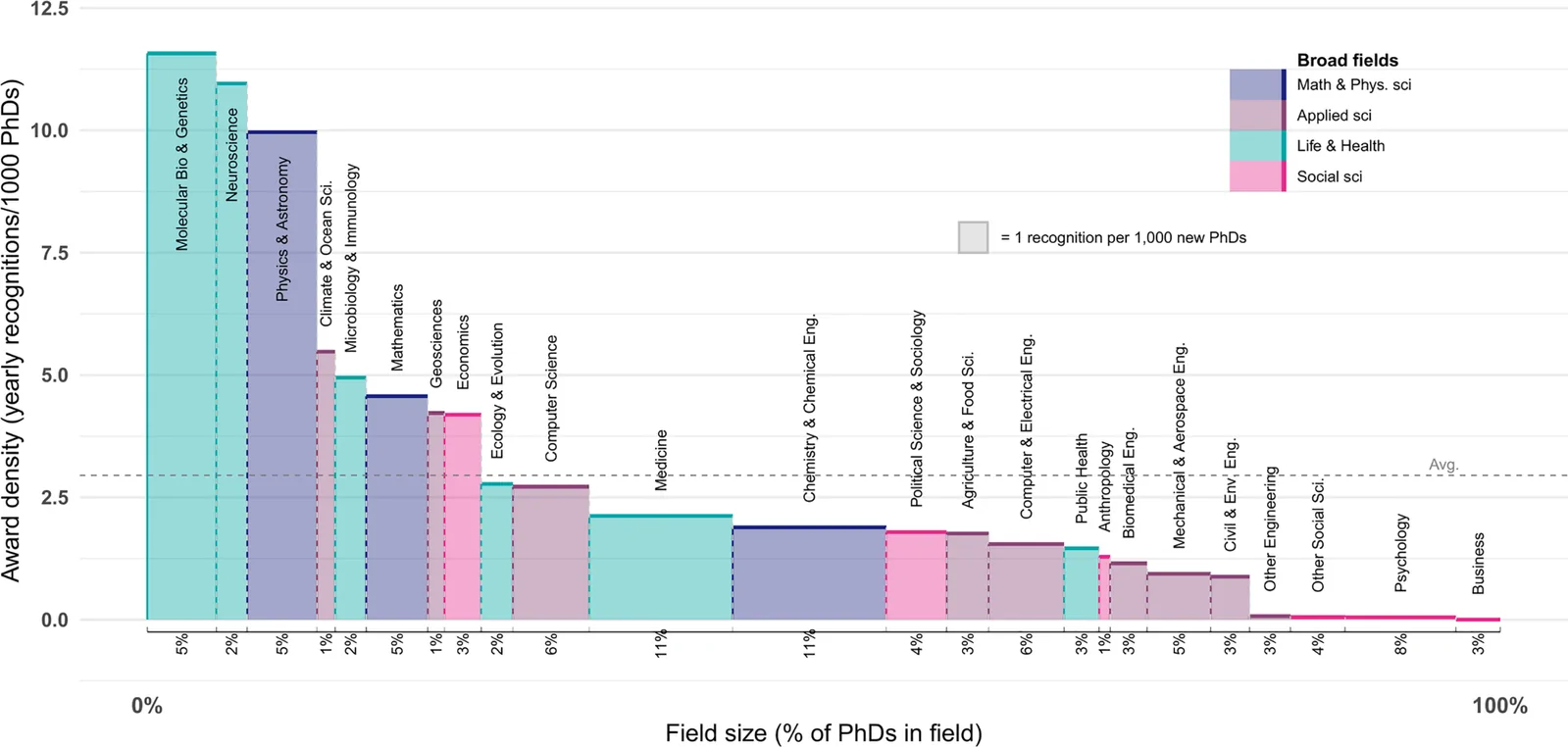
Award density by field (PhD denominator). *Note*: The figure plots the density of recognition events created by the 99 most prestigious prizes across 24 field groups (limited by NSF doctorate field classifications). Each recognition event is assigned to a field based on the winner’s primary research field (openalex.org author profile). Recognition events from 2015–2024 are averaged over 10 years. Field size is measured based on the number of new PhDs granted in the US in 2023. Density regions are colored by broad fields. The relative size of each field is displayed under each bar.

At the other end of the distribution, several large fields are consistently under-served regardless of the denominator. Computer science, despite its centrality to modern innovation, receives about 1.4 recognitions per thousand top academics—roughly one-quarter the density of physics—and ranks 10^th^ of 24 fields under the PhD denominator. Mechanical and aerospace engineering and civil and environmental engineering fall below one recognition per thousand academics and per thousand annual PhDs. Biomedical engineering is below one per thousand academics but reaches about 1.2 per thousand annual PhDs. Arguably, there is space for both general and specific engineering prizes. Although artificial intelligence is a rapidly growing field, no prize among the top 99 is specific to it.

Psychology and the social sciences excluding economics are underserved, regardless of the denominator used. Psychology, which accounts for 5% of academics at top universities and 8% of US PhDs, has no field-specific prizes; its low prize density derives entirely from unrestricted multidisciplinary awards. While psychology has some prestigious prizes in the US (e.g., awards from the American Psychological Association or the Society for Industrial and Organizational Psychology), these do not clear the bar in any of our inclusion criteria; the MacArthur fellowship, often awarded to psychologists, is only given to US residents, so it is excluded from our list. Political Science & Sociology and Anthropology also sit near the bottom under both measures.

Where the two measures of field size disagree, the discrepancies are informative. Chemistry ranks 4^th^ by academic density but drops to 12^th^ by PhD density, because chemistry departments train many doctoral students relative to their faculty size. Conversely, Economics ranks 14^th^ by academic density but rises to 8^th^ by PhD density, due to its large professoriate.

These inequalities become generally more accentuated if we restrict attention to the most prestigious prizes: Tier 1 prizes alone generate zero recognitions for most fields outside physics, mathematics, life sciences, and economics (S3 Fig in S1 Appendix), and including Tier 2 does not substantially change this pattern (S4 Fig in S1 Appendix). Dropping the prizes included only because of Criterion 3—those prizes that recognize mostly highly cited researchers, which could bias our list towards fields with more entrenched elites—decreases the inequality in field density, but not by much. The 23 prizes that enter the list through that criterion alone are all Tier 3 prizes, concentrated in the life sciences and astronomy. Dropping them reduces the Gini coefficient of field densities from 0.48 to 0.44 (per academic) and from 0.52 to 0.49 (per PhD). Using US federal research funding as the field-size proxy also places computer science and engineering and psychology below average, while other social sciences are above average under this measure (S1 Fig in S1 Appendix). Using the count of well-cited papers (at least 5 citations, 2018–2020) as the field-size denominator, which may better reflect the global amount of work in each field, yields the same broad pattern as our preferred US-based field-size measures (S5 Fig in S1 Appendix).

Proximity to first principles could explain why some fields receive more recognition. Comte’s hierarchy orders the sciences from most to least fundamental, with mathematics at one end and the social sciences at the other [34]. Recognition broadly follows this order, but differences within the biological sciences illustrate its limits: Microbiology & Immunology has the highest recognition density among the biological fields, whereas Medicine and Agriculture & Food Sciences rank much lower (Fig 3).

Similarly, prizes could be denser in more applied fields or in fields offering fewer market-based rewards. To assess proximity to application, we use the share of a field’s articles cited by US patents, drawing on Marx and Fuegi’s *Reliance on Science* data [35–37]. Across the 26 field groups, recognition density is *positively* associated with this share (Spearman ρ=+0.47, *p* = 0.016; S6a Fig in S1 Appendix), so fields that are more applied are relatively more recognized, not less. Economics and the social sciences cluster at low values of both measures; excluding them weakens the correlation to ρ=+0.15 (*p* = 0.519), but does not turn it around. To examine market rewards, we calculate median private-sector salaries of doctorate holders from the 2023 Survey of Doctorate Recipients public microdata, covering all career stages. Across 16 comparison groups covering 24 of our fields, recognition density is also positively associated with salary, although the estimate is imprecise (ρ=+0.43, *p* = 0.097; S6b Fig in S1 Appendix). Hence, although both comparisons rest on a rough classification of fields and may hide heterogeneity, neither supports a general pattern of prizes compensating for weaker market rewards.

### Career stages

Early-career prizes can function as both pull and push incentives, making them arguably more socially valuable per dollar than late-career awards. Yet only three among the 99 most prestigious prizes are early or mid-career prizes: the Fields Medal, the ICTP Ramanujan Prize, and the Max Planck-Humboldt Research Award (although the Maryam Mirzakhani Prize in Mathematics is described as a mid-career award, it imposes no clear age or career stage limit, and its 2020–2024 recipients were 15–25 years past their PhDs, so it does not fit our taxonomy). This scarcity of early career prizes could be rationalized if there were ample job and grant opportunities for young scientists [22,38,39], or if important discoveries were made only late in careers, but neither condition seems to apply [21,40].

To better understand this scarcity, and in an effort to increase the signaling value of these prizes, we also provide a list and ranking of early career recognition prizes (S9 Table in S1 Appendix). We construct this list based on prize age and online popularity, since surveys of experts did not include many early career prizes and information about prize money is often sparse for these awards (see S1 Appendix for details). Our strategy identifies the 68 most prestigious international early career prizes, which produce about 265 recognition events each year.

Four patterns in the early career list stand out. First, most of those prizes seem much less prestigious, or at least attract much less attention. The median early career prize draws 3.4 daily Wikipedia page views, against 13.9 for the median prize on the main list. Second, prestige concentrates in mathematics: nine of the ten Tier 1 early career prizes are mathematics prizes, although mathematics accounts for 35% of the prizes on the list and 10% of its recognition events. Third, there is much more heterogeneity in how many people are recognized by each award. On average, the early career prizes generate 1.6 times as many recognition events as the main list, but five cohort programs—the Sloan Research Fellowship (126 fellows a year), the three sections of the Max Planck Society’s Otto Hahn Medal, and the Amelia Earhart Fellowship—account for 72% of them, while 51 of the 68 prizes recognize at most one person a year. Fourth, the list spans several career sub-stages of what is understood as early career, revealing even more heterogeneity. Two-thirds of the prizes are open to established researchers, typically under 40 or 45 or within 10–15 years of the PhD, as with the COPSS Presidents’ Award and the Yrjö Jahnsson Award, while 12% go to doctoral students or recent graduates, such as the Otto Hahn Medal, and 19% to postdocs and new faculty, such as the Sloan Research Fellowship.

### Incentive design

Finally, we describe the magnitude and type of direct incentives that recognition prizes provide to their winners. There is substantial heterogeneity in how much money prizes distribute: eight prizes do not include a monetary value, and six prizes give out USD 1 million or more per winner. The median prize gives out USD 167,000 per winner (S7 and S8 Figs in S1 Appendix). Hence, most prizes pay their prizewinners less than what they would make in two years in their regular academic jobs. If we see prizes as a sort of performance bonus, these would be one or two orders of magnitude lower than top private market bonuses to CEOs [41].

Despite the high variance in money values, there is minimal variation in how the prize money is paid out. Out of the 91 prizes with an associated money value, only four have conditions on how the money is spent (the Balzan Prizes, which require half the award to fund projects by young researchers; the James Craig Watson Medal; the Max Planck-Humboldt Research Award; and the Tang Prize in Biopharmaceutical Sciences).

## Discussion

The recognition landscape is dynamic, but structurally incomplete. Half of the most prestigious prizes are less than 45 years old, showing that new awards can rise to prominence, but gaps persist, particularly in branches of engineering and of the social sciences, and at early career stages. In this section, we discuss prizes as signals and incentives, the possible welfare implications of our findings, and implications for designing and funding prizes in the future.

### Prizes as signals

#### Prizes as indicators of merit.

Our ranking measures prize prestige, but institutions often use prizes as evidence of research merit. Awards enter promotion criteria [42], and the European Research Council and NIH invite applicants to report honors and peer recognition [43,44]. University rankings and some talent-visa programs also rely on prizes [45–48]. How reliably prizes signal merit therefore matters for interpreting and using our ranking.

The selection of prizewinners can reflect factors beyond research merit. Historical studies document the role of political motives and nomination networks in Nobel selections during the twentieth century [2,11,12]. Campaigning for candidates and reciprocal favors among selectors could play the same role today, but without archival evidence for the contemporary prizes we study, we can only examine imbalances in recognition. In that vein, some studies have documented gender gaps in prize recognition and tested for bias in award selection [49,50]. Here, given the international character of our prizes, we use a geographic research benchmark to assess home bias as a potential indication of non-meritocratic influences.

We compare domestic recognition with the share expected from the geographic distribution of highly cited researchers in each field. Our list of prestigious prizes already excludes prizes with citizenship requirements, single-country winner histories, or no systematic selection procedure (Materials and methods). Nevertheless, across the 1,399 recognitions that prizes with a national awarding body made in 2015–2024 to laureates we could locate, 471 went to laureates based in the awarding country, against 336 expected if laureates were based where their field’s 1,000 most-cited researchers were in the award year. Domestic recognitions thus exceed the citation benchmark by 40%. For the citation benchmark, we count citations to papers with at most 20 authors to limit the influence of large consortia. Citations and affiliations are measured as of the award year. See the note to S9 Fig in S1 Appendix for details. Using as benchmark the top 0.1% or 0.05% of each field’s cited authors, so that benchmark size scales with field size, the ratio is 1.48× or 1.42×, against 1.40× with the top-1,000 benchmark. The extent of home bias varies across awarding countries and prizes (S9 and S10 Figs, S10 Table in S1 Appendix): Japanese awarding bodies recognize domestically based researchers at 7.5× their pool’s share, compared with 1.2× for US-based bodies; 67.7% of the 96 prizes exceed their benchmark.

These national home biases mostly offset in the aggregate (S3 Table in S1 Appendix, final three columns). Across all prizes, researchers based in the United States receive 65.0% of recognitions, close to their 65.0% share of the citation benchmark; the corresponding shares for the United Kingdom are 13.6% and 13.0%. For other countries, recognition departs from the benchmark in both directions: France receives 5.2% against 3.2%, while China, together with Hong Kong and Taiwan, receives 1.8% against 4.2%. A tendency to recognize domestic researchers therefore need not translate into an equally large imbalance in the aggregate geography of recognition, and on average these prizes may still be selecting meritorious researchers.

These comparisons show how the geography of recognition differs from that of citations; they do not identify the mechanisms behind the differences or test whether selection is fair. Citations have their own biases and reflect influences beyond conceptual significance [51,52]. Prize committees may also recognize qualities that citation totals miss: prizewinners produce more novel combinations of ideas than non-prizewinners with comparable publication and citation records [7].

Even if prizes are not pure indicators of merit, our list and ranking are helpful for two main reasons. First, for this study, the list and ranking allow us to characterize the landscape of prestigious recognition in science and identify gaps across fields and career stages. Second, our methodology can help decision-makers make better use of prizes as signals, as we give a transparent, empirical, and theoretically grounded basis for prestige comparisons. Because our data and methods are available and reproducible, decision-makers can make their judgments more explicit and open to scrutiny, as we exemplify below.

**Uses of the list and ranking.** Institutions using prize information could draw on this common scale to specify which awards they recognize and how much weight they give them. Next, we use the setting of skilled immigration policies to exemplify possible uses.

Several countries have skilled immigration policies in place, but all the cases we reviewed appear to under-use prize information. The UK Global Talent route uses an explicit list: holders of a listed prize can apply without an endorsement [47]. The list seems arbitrary: of the 73 prizes on the list examined for this study, 37 appear among our 99 most-prestigious prizes. The other 36 prizes on the UK list are mostly given by UK professional associations (22) and do not make it into our list for various reasons (e.g., five are in non-scientific fields, a few are given only to UK residents, others do not clear the bar in terms of our prestige indicators). Moreover, only six of the 73 prizes can be considered early- (or mid-) career prizes, and only one of those six, the Fields Medal, appears in our early career prize list. Other countries such as the US, France, and Australia are unclear or excessively restrictive about which prizes count. The US EB-1 category refers only to “a major, internationally recognized award” in its eligibility criteria [48]. Prize information could also be one input to points-based immigration systems, with points increasing by tier (our uncertainty estimates support comparisons across broad tiers more strongly than small differences in rank). The Canadian skilled immigration system, for instance, seems to completely disregard scientific prizes, prestigious or otherwise. Our prize rankings can be used to streamline procedures and provide clarity about which international prizes should be taken into account. If these “talent-visa” programs committed to a specific list of prizes, a significant source of uncertainty would vanish and prizewinners could become more willing to apply.

**Rank gaming and value capture.** These uses also carry risks. When a measure becomes a target, behavior may change in ways that weaken its informational value [53,54]. Nguyen [55] describes a related problem as value capture: a simplified measure can displace the underlying objective. For instance, decision-makers that need to evaluate research merit might instead simply look at how many prizes a certain researcher has. A ranking adds further risks: not only may prizegivers attempt to game it, but observers may also substitute simpler prestige comparisons for complex merit judgments. Besides cautioning readers about these risks, we hope to alleviate them by sharing our methods and data, making it easier for others to adapt them to their needs and to scrutinize them. We favor comparisons across broad tiers over small differences in rank, and the use of prize information alongside substantive assessment.

### Prizes and scientific progress

Besides providing signals, prizes create incentives. The prospect of status and monetary rewards can attract effort toward particular problems and fields [17–20]. Recognition may also reinforce inequalities or redirect effort away from more valuable work.

#### Effects of prizes on science.

Prizes can affect both recipients and the wider allocation of scientific effort. The literature points to three related effects. First, prizes can concentrate attention and resources among scientists who are already well recognized. In a network of roughly 3,000 scientific prizes, 64% of prizewinners receive more than one award, and recognition has become more concentrated over time [14]. Awards also increase the attention paid to winners’ earlier work [4]. Some of this attention comes at the expense of others: following an HHMI appointment, related papers by non-recipients lose citations relative to comparison papers [56]. These patterns are consistent with a “Matthew effect,” but need not imply a loss to science (e.g., awards may direct attention and resources toward more valuable work).

Second, recognition can affect winners’ research. Evidence on subsequent publication output is mixed across studies [5,57–59]. For instance, recipients of the John Bates Clark Medal publish more after the award relative to a synthetic comparison group [5], while Fields medalists publish less but explore more new topics, relative to other leading mathematicians [59].

Third, prizes can attract researchers to a field. Jin, Ma and Uzzi [3] find that prizewinning topics subsequently attract more entrants and produce more papers and citations than topics with similar prior growth. Hence, prizes could change the allocation of scientific effort beyond their effects on individual recipients. Whether this redirection is a net benefit depends on whether prizes reveal useful information (e.g., about the future social importance of a field) or not.

Taken together, these studies show that prizes can have consequences for winners and their fields, but do not establish their net effect on scientific progress. In particular, the studies we review examine recognition by existing awards, not the effect of creating a new prize relative to using the same resources elsewhere. The design proposals below should therefore be read as possible ways to improve recognition, not as evidence that expanding the prize system would increase welfare.

#### Do field coverage gaps matter?

Prizes complement rather than replace grants and other forms of scientific support. As fields may differ in their reliance on prizes relative to other incentives (and in the value that each incentive generates), the inequality in the recognition provided by prestigious prizes by itself does not imply that fields are providing too few or too many incentives to their scientists. The positive or null associations between market rewards and prize density reported in the Results section suggest that the market does not supply these alternative rewards, but compensation could still occur in other ways.

Because prizes are widely legible (e.g., in comparison to grants), prizes produce information reaching beyond the realm of current practitioners. In this case, inequality in field coverage can be particularly relevant. As discussed above, the uses of prizes in hiring, funding and immigration make their distribution consequential even when their net welfare effects are uncertain. Three further channels may be relevant. First, the legibility of prestigious prizes allows them to be used as a tool to display canonical, key findings to the public, increasing the salience of a scientific consensus. In fields without prestigious prizes, it may be harder for the public to distinguish the consensus from the fringe. Second, prizes can raise the status of a field and lead the young to pursue scientific subjects. Uneven recognition may therefore affect which fields provide visible role models. Third, scientists across disciplines can also benefit from richer prize signals. For instance, if prizes in an adjacent discipline highlight a useful methodology, that can lead to new breakthroughs in other fields. Krauss [16] documents the importance of methods developed in other disciplines for Nobel-recognized breakthroughs in economics, illustrating the potential value of such diffusion. These channels motivate attention to the gaps; they do not establish that equal coverage would be optimal. Of course, if the value of legibility outside the field varies across fields, the inequality in coverage may not imply an inefficiency.

### New and better prizes

As prize creation remains a popular endeavor, we now discuss the implications of our findings for future prize design. Conditional on funding a prize, a funder can seek to preserve its informational benefits while addressing specific limitations, such as late recognition or the exclusion of contributors. These changes involve trade-offs, which must be evaluated case by case. We start by describing how the most prestigious prizes have been created, which frames the discussion.

Recognition prizes have been a mostly private endeavor, and they are a relatively cheap part of the system of incentives in science. Philanthropic or corporate money financed 75% of the prizes founded before 1980, 82% of those founded in 1980–1999, and 77% of those founded since 2000 (S11 Fig in S1 Appendix). In total, our prestigious prizes distribute about USD 64 million annually, 0.03% of US federal R&D obligations [60]. Hence, there is no major trade-off between the public funding of science and the funding of recognition prizes. Private interests may seek to manipulate scientific progress by funding fields that are closer to market applications, but we do not see correlational evidence for that. Weighting by recognitions in 2015–2024, the share of articles cited by patents in the winners’ fields is 7.7% for prizes whose purse is privately funded in the latest award cycle and 7.9% for other prizes. Prestigious prizes also show no tendency of becoming privately-funded later in their histories as, on net, among the prizes whose funder changed, the number of privately funded ones decreased by two.

The prizes on our list were created when their fields were already well-established: even the quickest arrived a decade or two after their fields formed. The Tyler Prize (1974) came nine years after the first environmental studies degree program, the Von Hippel Award (1976) eighteen years after the first materials science department, the Kavli Prize in Nanoscience (2008) eighteen years after the field’s first journal, and the Turing Award (1966) nineteen years after the Association for Computing Machinery was founded. A young field is therefore recognized, when it is, by the prizes of older fields whose scope covers it, as artificial intelligence is by the Turing Award. A field that falls outside the scope of every existing prize may wait decades for one of its own. Founders of new prizes may look for such fields, or design their prizes flexibly enough to allow the recognition of new rising fields.

Case studies of successful new prizes show that, even recently, it was possible to create “Nobels” for fields without them. For instance, the Queen Elizabeth Prize for Engineering (26^th^ in our ranking) and the Abel Prize in Mathematics (7^th^) were both established in the 2000s. Both prizes were created with government backing, the less common route, and each offers substantial prize money (the Abel Prize awards NOK 7.5 million, roughly USD 750,000; the Queen Elizabeth Prize awards GBP 500,000, roughly USD 650,000). Hence, given enough resources and institutional credibility, it is possible to create successful, Nobel-like prizes. Nevertheless, not all fields have succeeded in creating Nobels. In psychology, no field-specific international prize meets our inclusion criteria, leaving a gap that prospective funders could consider. Although we do not include prizes in the field of education in our list of top prizes, the case of the Yidan Prize in Education shows what is possible. The Yidan prize was first given in 2017, but already has some notable prestige and visibility, with 15 daily page views, and no obvious close competitor in its field. It also presents its winners with prize money and R&D funds averaging about four million dollars per year.

Next, we consider early career prizes. The Fields Medal, given to mathematicians under 40, is the only early career prize in the top tier. Most other fields lack a highly prestigious early-career award, but we see at least three pathways to fill those gaps. First, it is possible to create completely new prestigious early career prizes; the New Horizons prizes in Physics and Mathematics (first given in 2013 and 2016) demonstrate this path, drawing funding and visibility from the Breakthrough prize family and offering USD 100,000 per prize, which may be shared by multiple recipients. Second, it is possible to convert already-prestigious prizes into early career prizes, as shown by the transformation of the Max Planck Research Award into the Max Planck-Humboldt Research Award, which now targets researchers within 15 years of their PhD and offers 1.5 million euros in research funds. Third, existing early-career prizes are often underfunded and can benefit from more support; strikingly, the Fields Medal, which we rank 5^th^ among all prizes in prestige, offers only 15,000 Canadian dollars in prize money. Using this strategy, funders may be able to raise existing early career prizes to higher levels of prestige relatively quickly, as many underfunded prizes already have a history of excellence.

One difficulty in establishing prestigious early career prizes is that top contributors are hard to identify early enough. Nine of the ten Tier 1 prizes in our early career list are in mathematics. This concentration is consistent with early contributions being easier to identify when they are self-contained and attributable to individuals, which may be harder in more multi-disciplinary fields. Nevertheless, cohort-based awards show that it is possible to recognize large numbers of individuals over several fields: for instance, the Sloan Fellowship, which accounts for 48% of the recognitions created by prizes in our early career list, is awarded to multiple economists, neuroscientists, and molecular biologists every year.

A further margin is how many people a prize recognizes. Science is increasingly done in teams, particularly its highest-impact work [61], yet the 784 awards in our sample named 1.9 individuals on average, and 29 of the 99 prizes never divided an award during 2015–2024. Limits on recipients can exclude contributors to joint discoveries [62,63] and may weaken incentives to collaborate [64,65]. Recognition can also change the subsequent allocation of credit: after a Nobel award, citations to the prizewinning work become more closely associated with the laureate than with coauthors [62]. Funders could consider recognizing teams or named contributions, without requiring committees to allocate joint credit to a few individuals. Wider recognition is feasible: the Breakthrough Prize in Fundamental Physics has honored the 347 members of the Event Horizon Telescope collaboration (2020) and the three LIGO founders together with 1,012 further contributors (2016), and the Royal Society’s 2022 Copley Medal went to the Oxford–AstraZeneca vaccine team. In our data, prestige is positively correlated with recipients per award (*r* = +0.29, *p* < 0.01), so, although increasing the number of winners may dilute a prize’s prestige, prizes that name more people can still rank among the most prestigious.

Finally, prizegivers interested in accelerating scientific progress could complement recognition with incentives for subsequent work. While over half of top CEOs’ compensation comes in the form of stock and options [41], most recognition prizes do not tie payments to subsequent research. Grants can support future work, but evidence points to limits on their capacity to expand [66] or redirect [67] research output. Fellowship-style awards that combine recognition with multi-year research support, such as the Sloan Fellowships or HHMI Investigatorships, offer a possible template. Funders may also incentivize applications of certain discoveries. For instance, a prize for new therapies could include an incentive akin to an advance market commitment, in which the prizegiver promises in advance a reward if the therapy meets actual market demand; prizewinners would then have an incentive to direct their research toward guaranteeing that a technology reaches the public [24]. Mentorship of younger scientists could also be incentivized, either similarly to the Balzan prizes or in the form of bonuses for supervising dissertations. Such conditions may encourage follow-on work but also redirect effort toward easily measured outcomes, which may not be optimal, so these rewards need careful design.

## Conclusions

The gaps we document describe the distribution of prestigious recognition, not an optimal allocation of rewards. Subject to the caveats discussed above, our analysis points to three design principles for future scientific prizes. First, recognition could adapt to the structure of modern science. Breakthroughs increasingly arise at the intersections of established disciplines, yet most prizes remain tied to legacy field boundaries. Future prizes could rotate across emerging fields, allowing prestige to track where discovery actually occurs, and recognize joint contributions more broadly.

Second, prizes could accelerate future work, not merely commemorate past achievement. Early- and mid-career scientists stand to gain the most from recognition, yet almost no top prizes target them. Linking awards to research continuation grants, mentorship, or milestones toward practical use could shift prizes from one-time honors toward sustained support.

Third, coverage could broaden. Engineering, computer science, psychology, and the social sciences remain underrepresented among top prizes, as do scientists working outside traditional research powerhouses. Philanthropists and institutions creating new prizes could address these gaps directly—and our data show that doing so is feasible, since half of today’s most prestigious awards are less than 45 years old.

## Supporting information

S1 Appendix Supplementary figures and tables, and early career prize methodology. S1–S11 Figs, S1–S10 Tables, and the construction of the early career prize list.
(PDF)
