# Supplementary material for: The missing Nobels: Mapping the landscape of prestigious scientific prizes
Source: PLoS One. 2026 Nov 13;21(11):e0361810. doi: 10.1371/journal.pone.0361810 (PMC13766277; doi:10.1371/journal.pone.0361810)
Supplement: Supplementary file 1 [file pone.0361810.s001.pdf]

S1 Appendix

The “Missing Nobels”

Supplementary figures and tables

Fig S1. Award Density by Funding

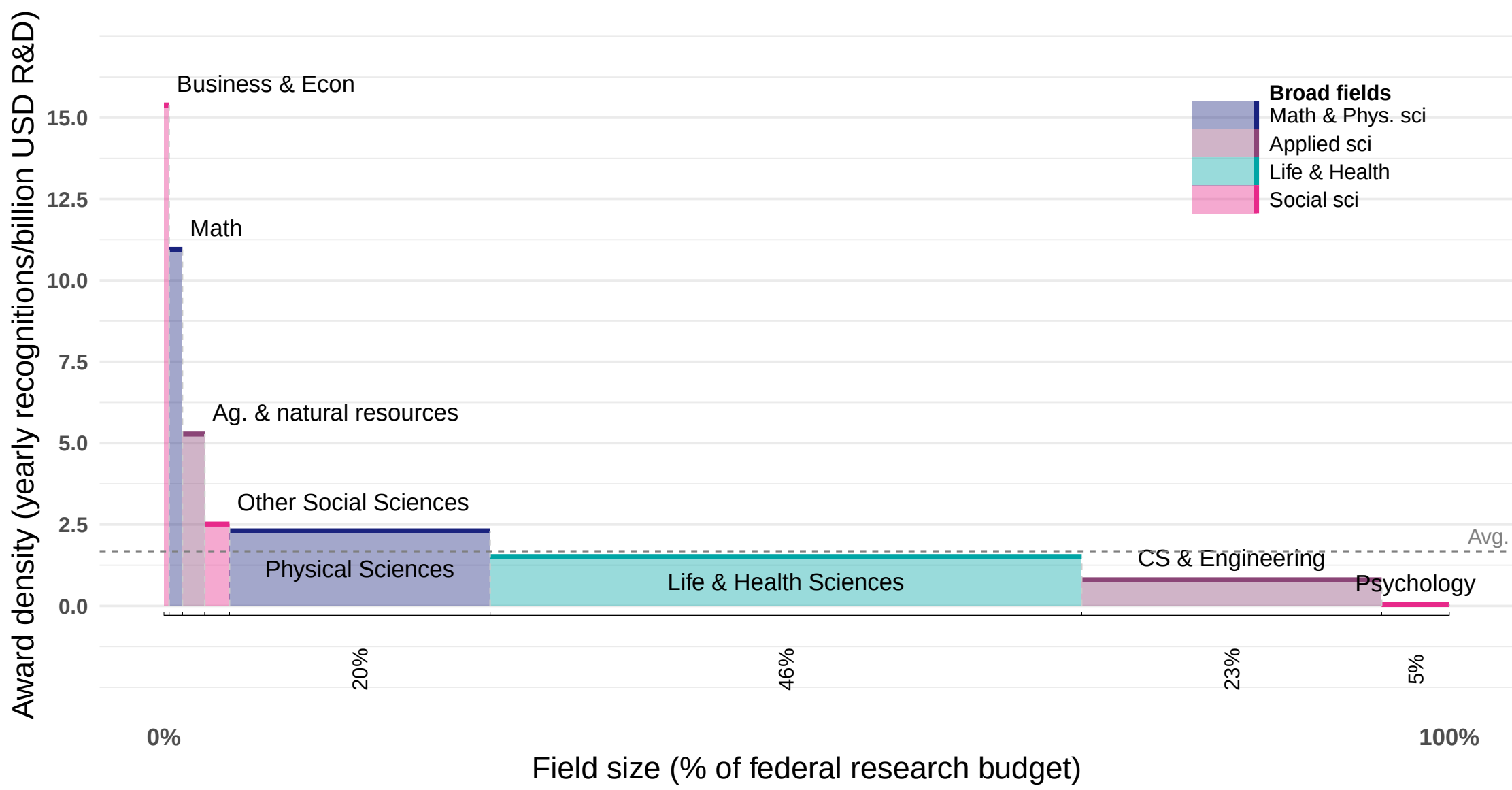

Note: The figure uses observed recognitions in 2015–2024, assigned by winners’ OpenAlex fields to eight funding categories and averaged over ten years. Density is yearly recognitions per billion US dollars of federal research funding in 2022. The 98-prize sample excludes the discontinued Max Planck Research Award, the predecessor of the included Max Planck–Humboldt Research Award; records without a mapped scientific subfield are excluded. Field size is each category’s share of the included research budget. Density regions are colored by broad fields. The relative size of each field is displayed under each bar.

Fig S2. Unconditional Correlation Between Purse Value and Online Visibility

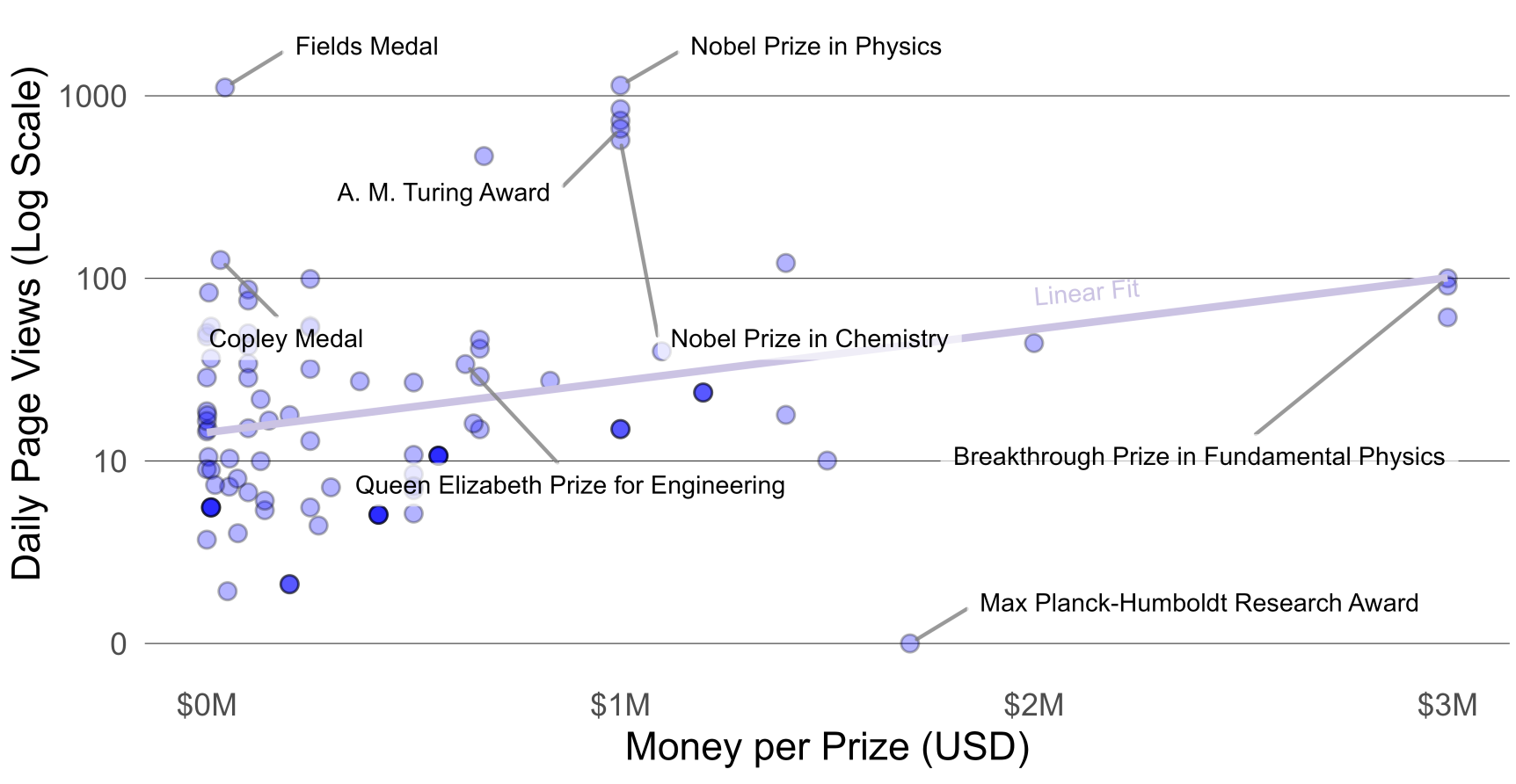

Note: The figure shows the relationship between daily Wikipedia page views (vertical) and prize money per award, which may be shared among multiple winners (horizontal), for 98 prizes. The discontinued Max Planck Research Award is excluded as the predecessor of the included Max Planck–Humboldt Research Award.

Fig S3. Award Density by Field: Tier 1 Prizes Only

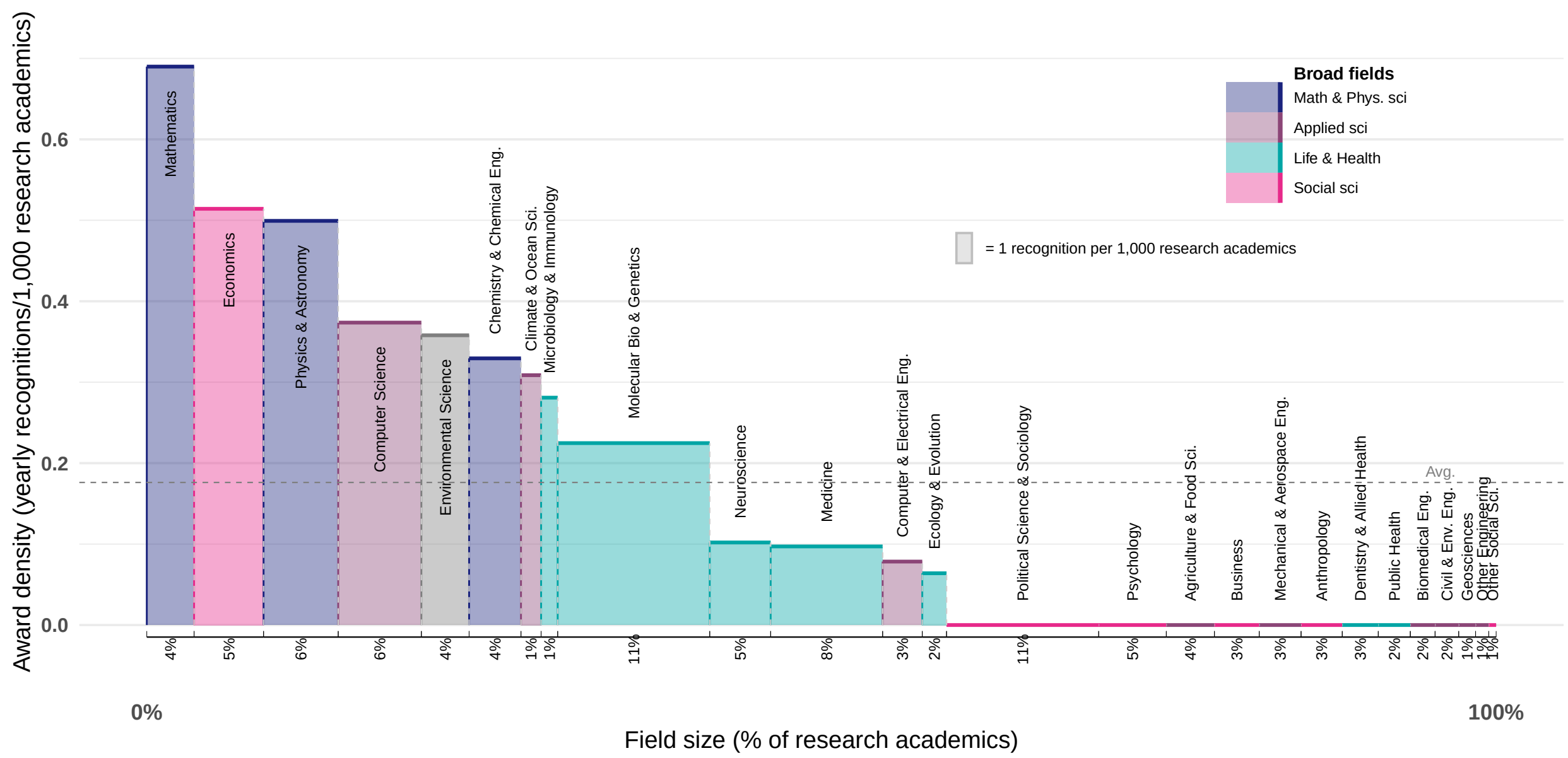

Note: The figure plots the density of recognition events created by the eight Tier 1 prizes across 26 field groups. Each recognition event is assigned to a field based on the winner's primary research field (openalex.org author profile). Recognition events from 2015–2024 are averaged over 10 years. Field size is measured based on the number of research academics at the top 150 US universities. Density regions are colored by broad fields. The relative size of each field is displayed under each bar.

Fig S4. Award Density by Field: Tier 1 and Tier 2 Prizes

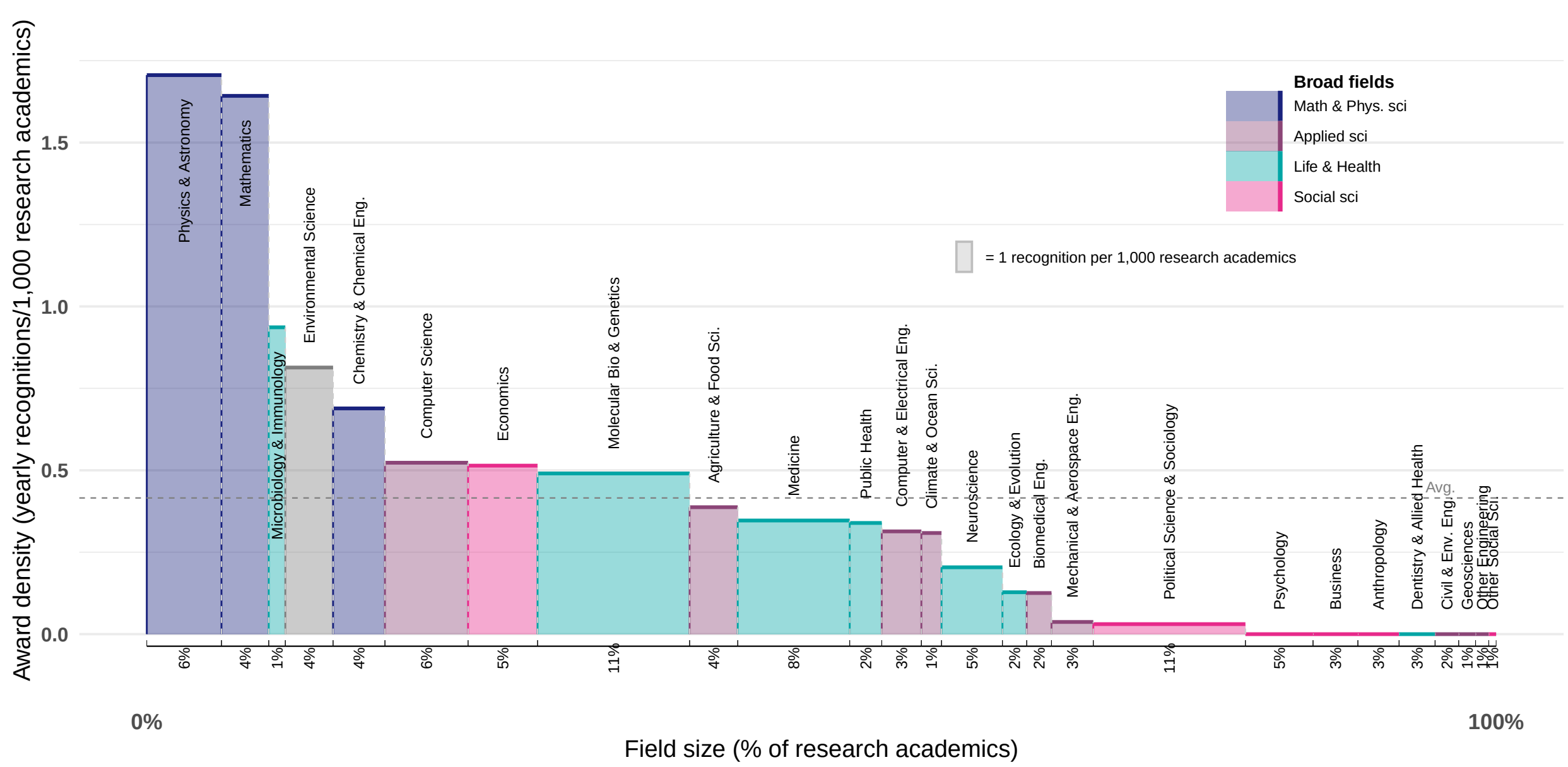

Note: The figure plots the density of recognition events created by the 20 Tier 1 and Tier 2 prizes across 26 field groups. Each recognition event is assigned to a field based on the winner's primary research field (openalex.org author profile). Recognition events from 2015–2024 are averaged over 10 years. Field size is measured based on the number of research academics at the top 150 US universities. Density regions are colored by broad fields. The relative size of each field is displayed under each bar.

Fig S5. Award Density by Field (Well-Cited Works Denominator)

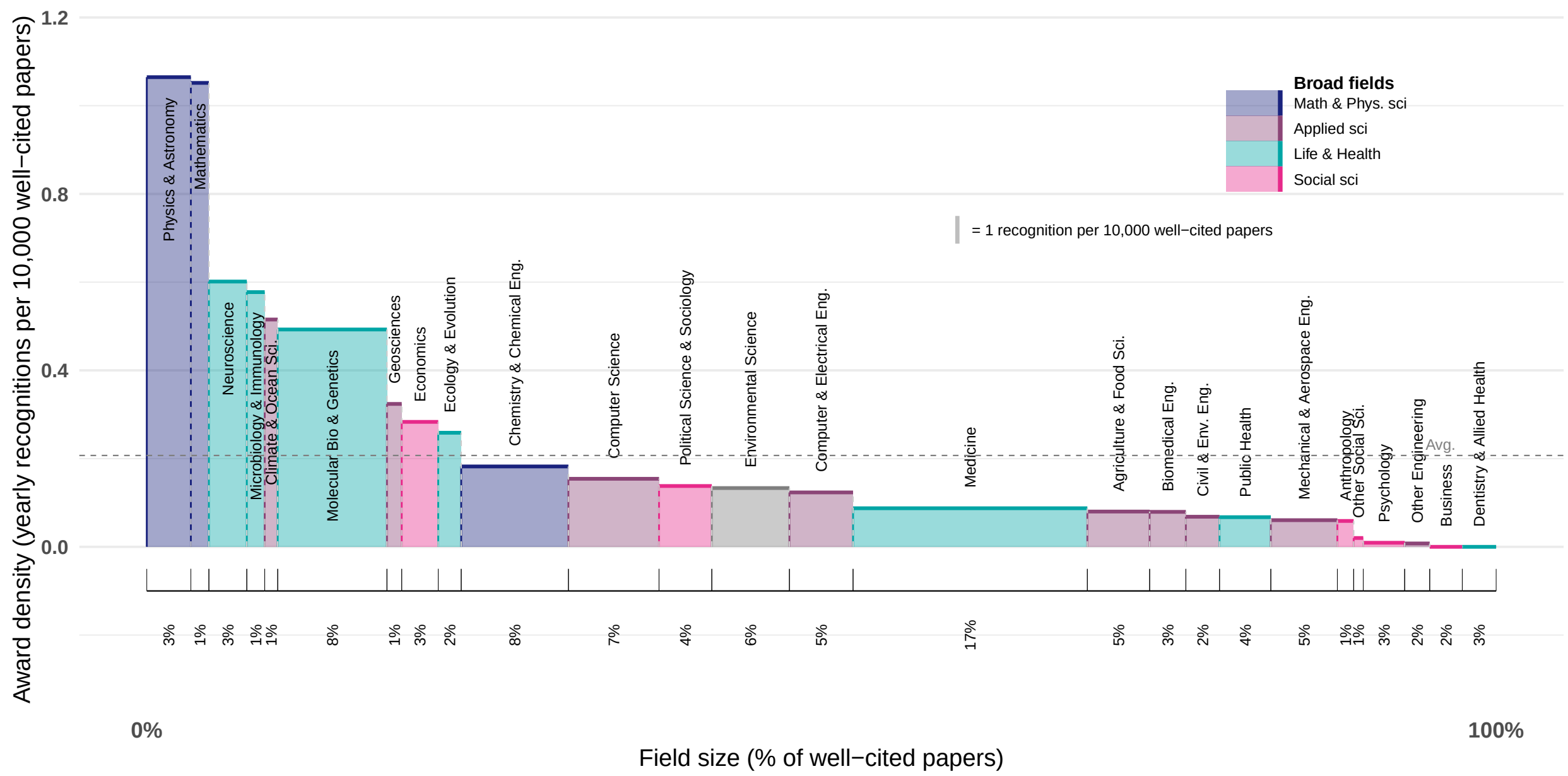

Note: The figure plots the density of recognition events created by the 99 most prestigious prizes across 26 field groups. Each recognition event is assigned to a field based on the winner's primary research field (openalex.org author profile). Recognition events from 2015–2024 are averaged over 10 years. Field size is measured by the number of OpenAlex works with at least 5 citations published in 2018–2020. Density is expressed as yearly recognitions per 10,000 well-cited papers. Density regions are colored by broad fields. The relative size of each field is displayed under each bar.

Fig S6. Prize Density, Patent Citations, and Private-Sector Pay

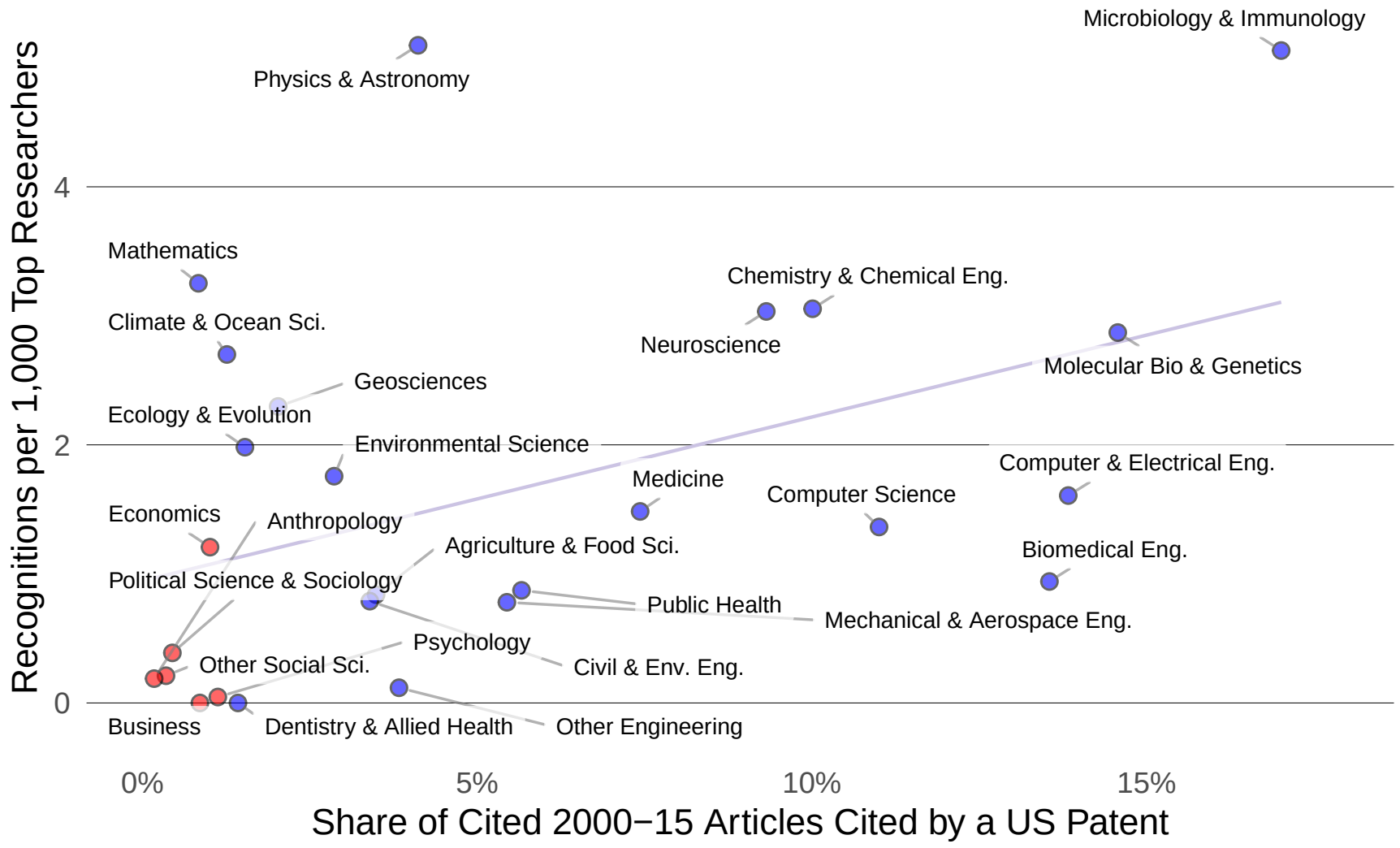

(a) Patent citations

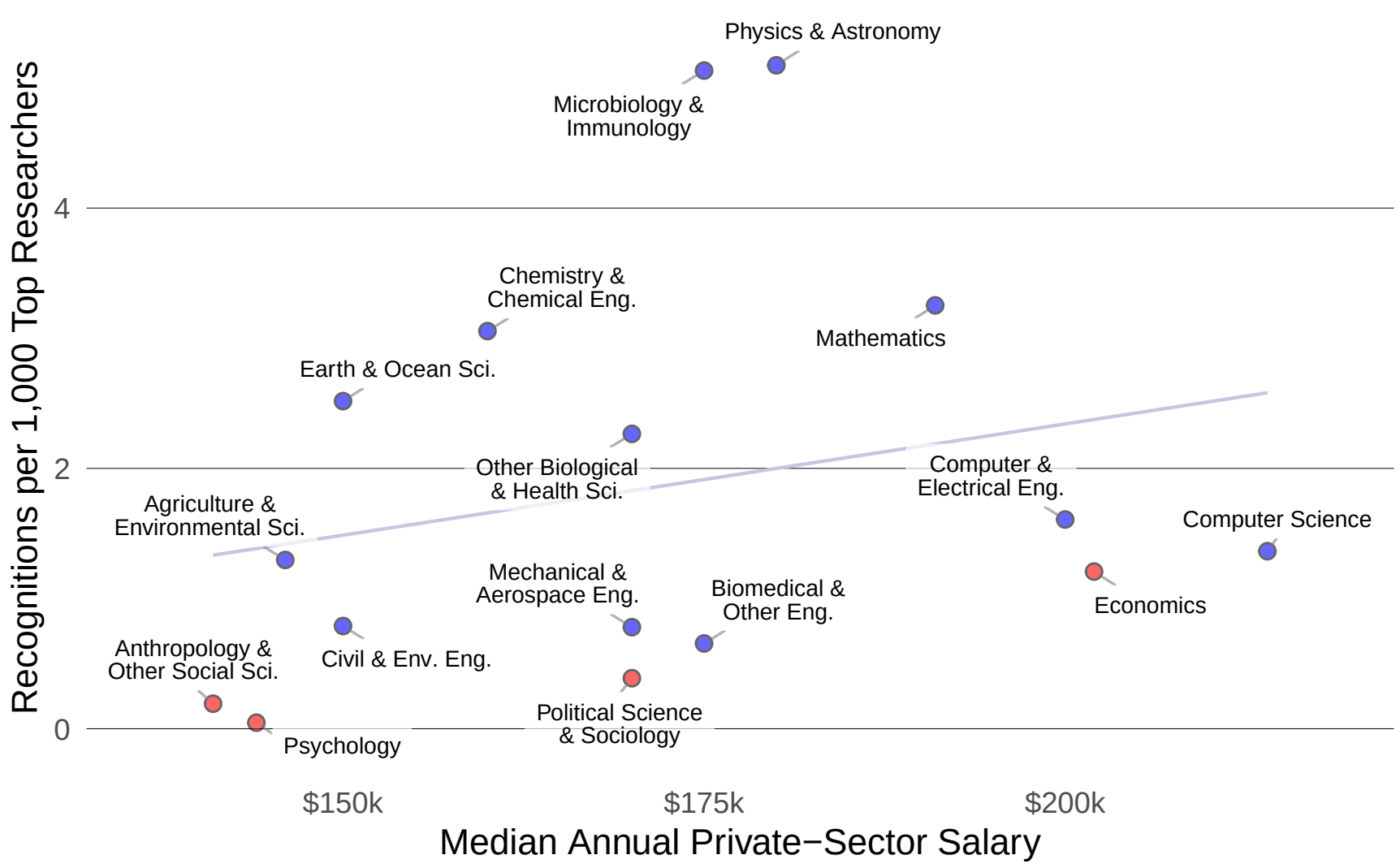

(b) Private-sector pay

Note: Both panels plot yearly recognitions per 1,000 research academics, 2015–2024, as in Fig 3; lines are linear fits; social-science fields are red. Panel (a): share of a field's 2000–2015 articles with at least one scholarly citation in OpenAlex (32.6 of 86.9 million, assigned to fields by OpenAlex primary topic) that a US patent granted through 2025 cites, from the July 2026 release of Marx and Fuegi's *Reliance on Science* [1–3], counting front-page and in-text citations with confidence at least 4 on the authors' 1–10 scale. Restricting to cited articles limits uneven inclusion of never-cited records across fields; the window allows at least ten years for a patent citation. Panel (b): survey-weighted median annual salary of US-residing doctorate holders working over 35 hours per week in private for-profit employment (incorporated self-employment included), all occupations, from the survey's public-use file. The survey's field categories are broader than ours, so we pool fields to match them: Agriculture & Food Science with Environmental Science; Geosciences with Climate & Ocean Sciences; Anthropology with Other Social Sciences; Biomedical with Other Engineering; and Molecular Biology & Genetics, Neuroscience, Ecology & Evolution, Medicine and Public Health into one biological and health sciences group. Business and Dentistry & Allied Health have no survey counterpart and are omitted.

Fig S7. Prize Money per Winner: Distribution Across Prizes

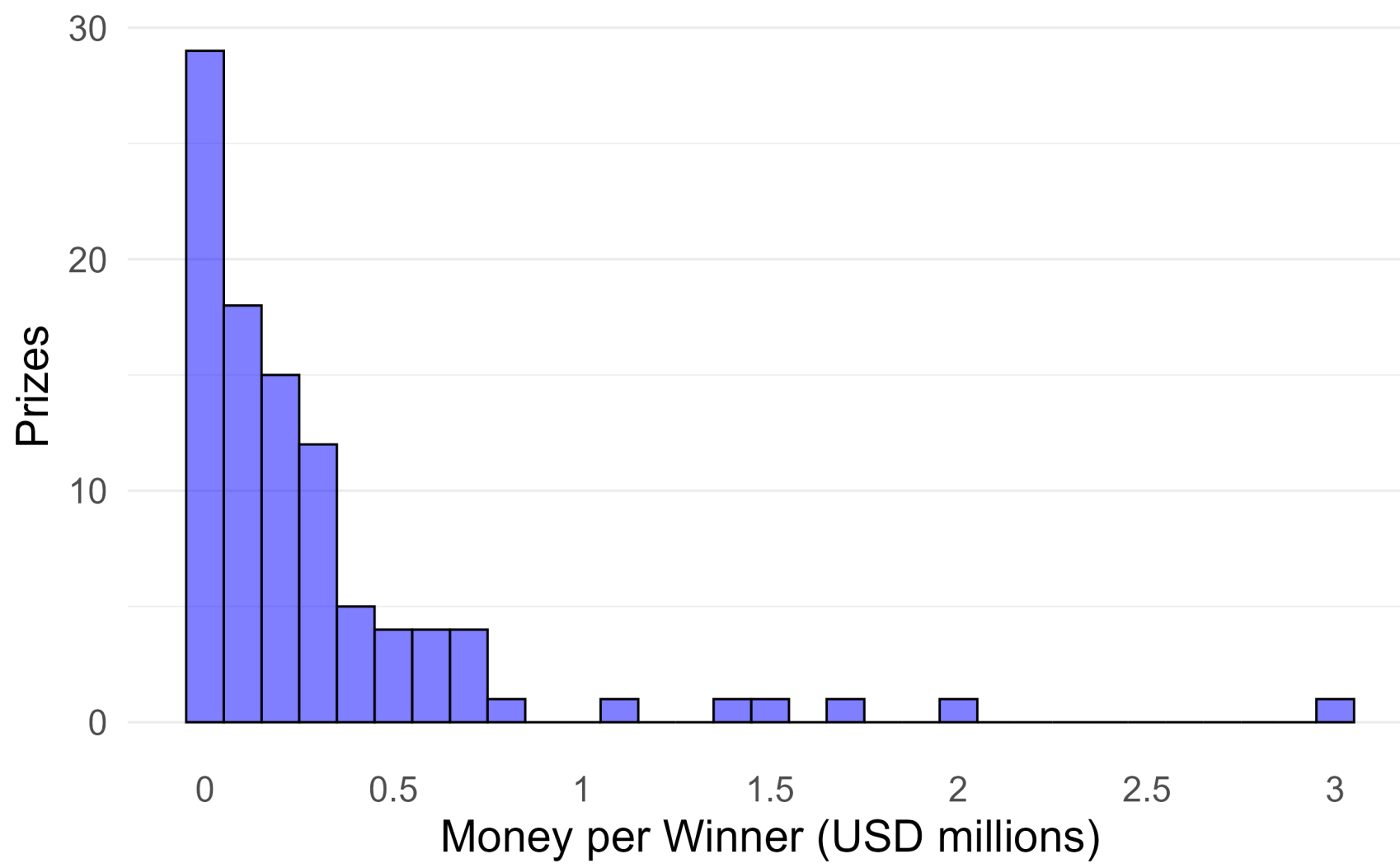

Note: The figure counts prizes in bins of money per winner. Each of the 98 included prizes has equal weight. The discontinued Max Planck Research Award is excluded as the predecessor of the included Max Planck–Humboldt Research Award.

Fig S8. Prize Money per Winner: Weighted by Annual Recipients

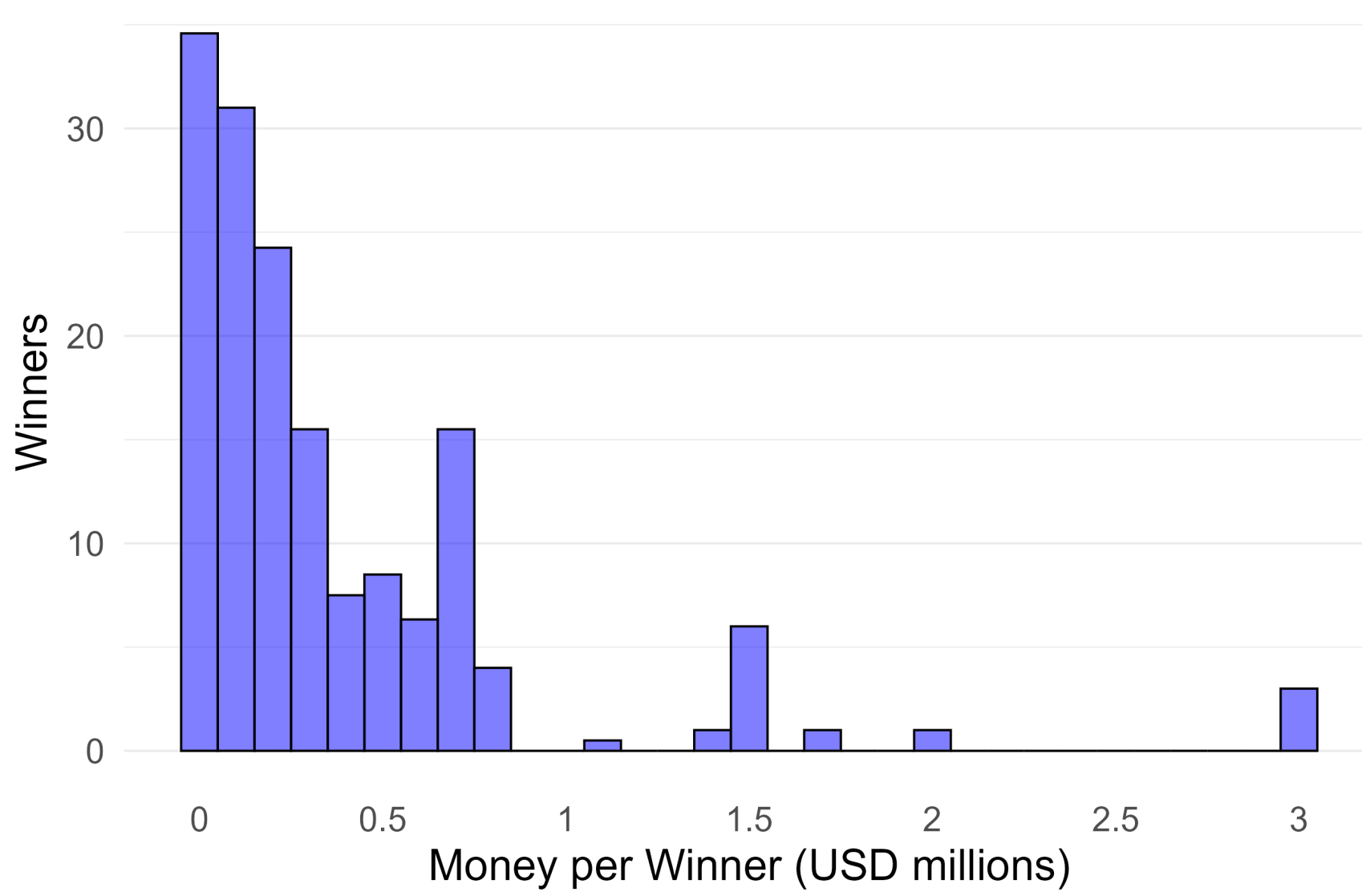

Note: The figure shows binned money per winner, weighting each prize by its estimated number of annual recipients. The 98 included prizes exclude the discontinued Max Planck Research Award, the predecessor of the included Max Planck–Humboldt Research Award.

Fig S9. Home Recognitions by Awarding Country, 2015–2024: Actual Versus Expected Share

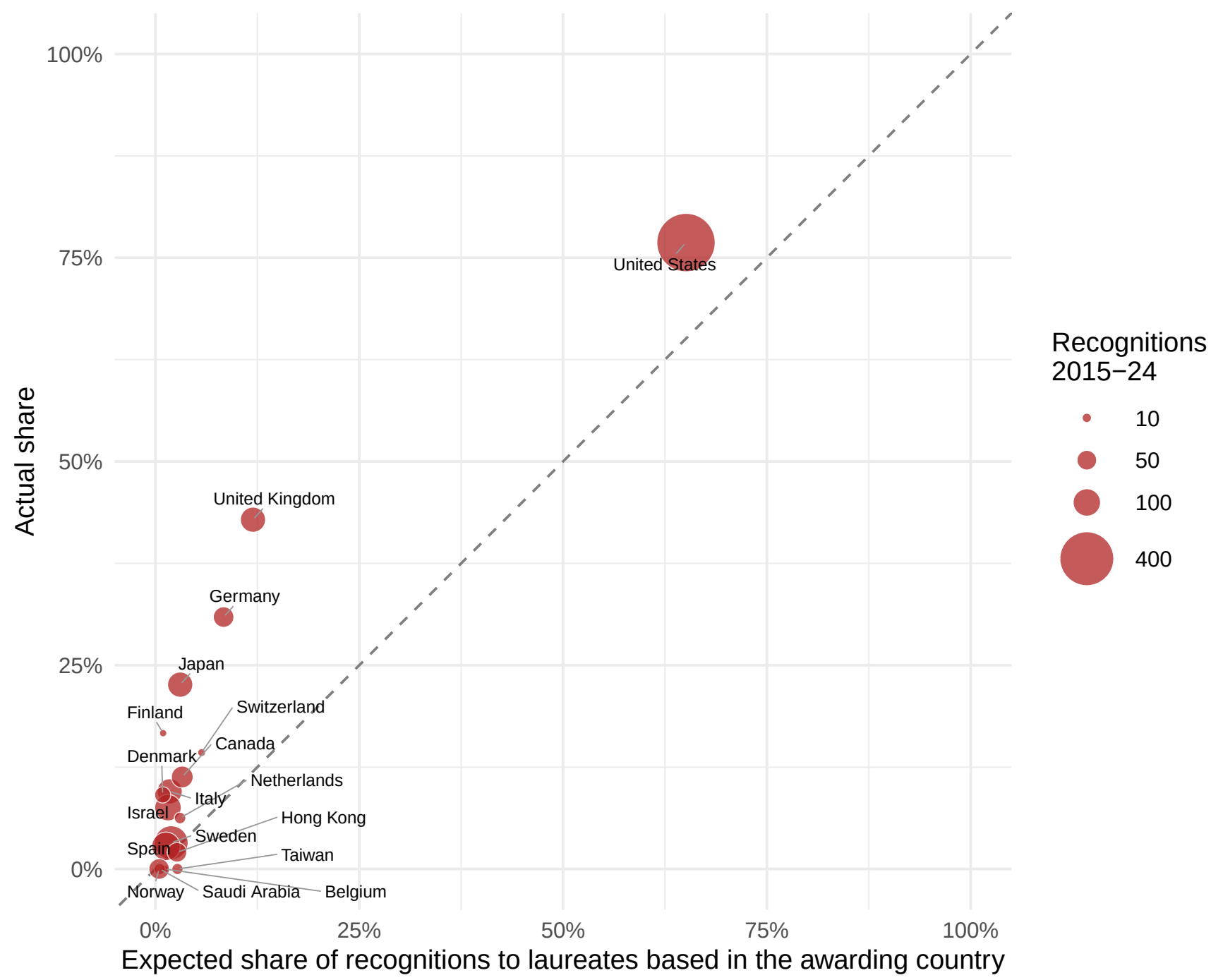

Note: Each point is an awarding country, sized by its number of recognitions. Horizontal axis: the share of its prizes' recognitions expected to go to a laureate based in that country under the benchmark; vertical axis: the share that did. Countries above the dashed line recognize home-based laureates more often than the benchmark predicts. Benchmark: for each field and award year, the 1,000 researchers with the most citations, as of the award year, to papers with at most 20 authors (small-team papers), which limits the influence of large consortia in physics and medicine. Laureates and benchmark researchers are located through the institutions and countries on their small-team papers; an affiliation counts if it appears in at least two publication years up to the award year and on at least 5% of the author's small-team papers, which limits errors from shared affiliation lists and mismatched institutions.

Fig S10. Home Share of Each Prize's Laureates Against the Field Benchmark

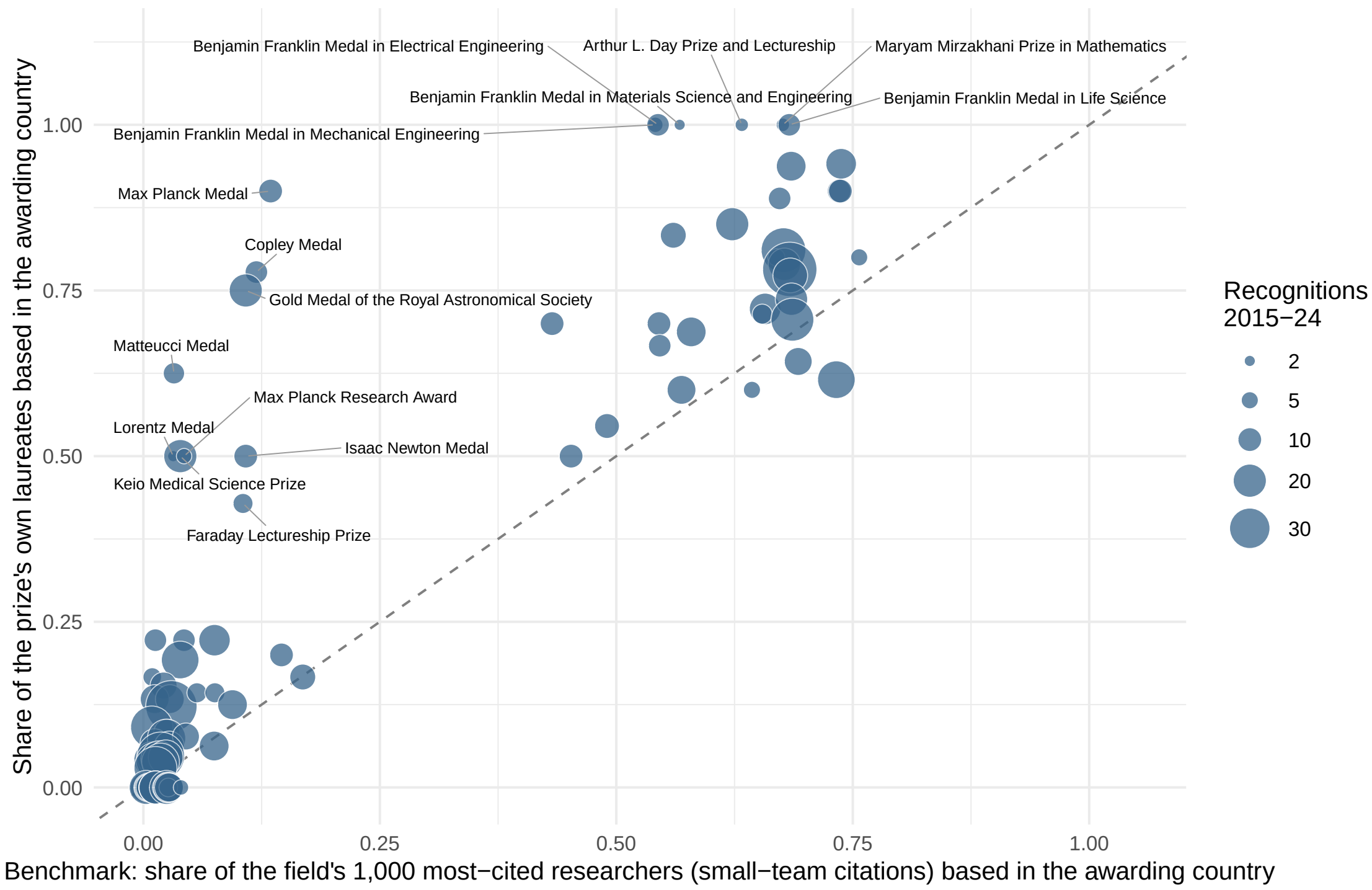

Note: Each point is a prize with a national awarding body, sized by its number of recognitions. Horizontal axis: the share of the benchmark researchers (note to Fig S9) based in the awarding country in the prize's award years; vertical axis: the share of the prize's own 2015–2024 laureates based there. Prizes above the dashed line recognize home-based laureates more often than the benchmark predicts. Labels mark prizes more than 30 points above or 15 points below the benchmark. Prizes with few recognitions scatter widely, since one laureate moves their home share by a large fraction.

Fig S11. Prestigious Prizes by Funder Type at Founding, 1900–2025

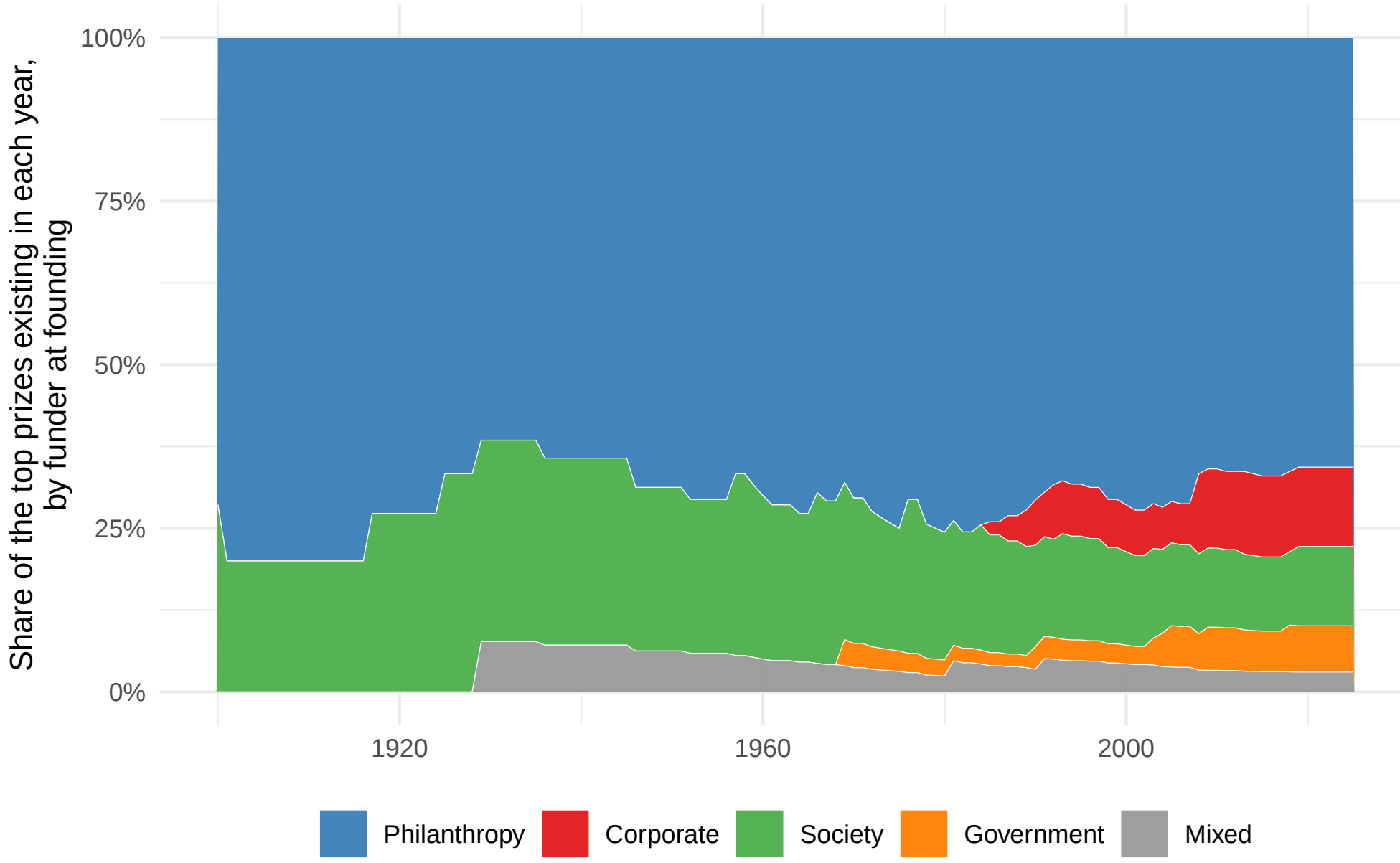

Note: For each year, the figure splits the prizes already founded by the type of source that supplied more than half of the purse at founding (mixed when none did). Philanthropic and corporate funders are the top two bands, so together they give the privately funded share; no prize was founded with university money. The funder is whoever pays the purse (prize money and any research funds to the winner; the medal when there is no cash), at founding or at the last refounding with new money. A language-model agent coded each prize under a written protocol from the prize's site, the awarding body's reports or filings, and press announcements (Wikipedia only as a supplement), recording its sources and the passage that establishes the funder; where an alternative coding is defensible, the record flags it and states why we chose ours.

Table S1. Lists of Prestigious Awards

List	Compilers	Number of Prizes	Year
US National Research Council's list of prestigious awards for ranking doctoral programs	Domain experts	191	2006
Inventory of International Awards	Government of Canada	135	2018
Roster of Distinguished Awards	International Congress of Distinguished Awards	95	2014
Prizes known as the Nobel of a field	Wikipedia contributors	63	2019
Prizes considered in the CWUR World University Rankings	CWUR	30	2019
Prizes considered in the Shanghai University Rankings	ARWU, based on their survey of academics	26	2019
Major prizes for medical research	Naylor and Bell (2015, JAMA)	20	2015

Note: This table summarizes the seven lists of prestigious prizes used in selection Criteria 1 and 2.

Table S2. Inclusion Criteria: Prizes Meeting Each Criterion and Prizes Meeting No Other

Inclusion criterion	Prizes meeting it	Meeting no other
1. Appears on at least three of seven authoritative lists	42	5
2. Expert rating ≥ 0.5 of a Nobel, and on at least one list	55	11
3. Over 50% of recent winners on Clarivate's HCR list	53	23
4. At least 40 daily Wikipedia page views, 2020–2025	26	6
At least one criterion	99	
Two or more criteria	54	
All four criteria	4	

Note: A prize enters the list if it meets at least one criterion; the citation criterion is evaluated over prizes that appear on at least one of the seven authoritative lists or were rated in either expert survey.

Table S3. Awarding Countries of the 99 Prizes, Home Bias, and Where Laureates Are Based

Country	Prizes given by country	Recognitions awarded by the country's organizations		Share of the country's recognitions given to scientists based there (%)			Share of all recognitions received by scientists based in the country (%)		
		N	% of all	Actual	Expected	Ratio	Actual	Expected	Ratio
United States	33	485	33.2	76.9	65.1	1.2	65.0	65.0	1.0
Sweden	13	162	11.1	3.2	1.9	1.7	2.0	1.9	1.1
Spain	5	109	7.5	2.8	1.3	2.2	0.6	1.2	0.5
Israel	6	93	6.4	7.5	1.5	4.9	2.2	1.5	1.4
Italy	3	90	6.2	9.5	1.7	5.5	1.8	1.9	0.9
United Kingdom	7	87	6.0	42.9	12.0	3.6	13.6	13.0	1.0
Japan	5	85	5.8	22.6	3.0	7.5	4.4	3.4	1.3
Canada	2	62	4.2	11.3	3.3	3.4	4.6	4.3	1.1
Germany	5	56	3.8	30.9	8.4	3.7	10.0	8.1	1.2
Norway	4	55	3.8	0.0	0.4	0.0	0.1	0.4	0.3
Hong Kong	3	50	3.4	2.0	2.6	0.8	0.4	0.7	0.6
Denmark	1	33	2.3	9.1	0.9	10.3	1.5	1.2	1.3
International	3	26	1.8	–	–	–	–	–	–
Netherlands	4	16	1.1	6.2	3.0	2.1	1.7	2.8	0.6
Saudi Arabia	1	16	1.1	0.0	0.5	0.0	0.4	0.6	0.7
Taiwan	1	15	1.0	0.0	2.7	0.0	0.0	0.3	0.0
Switzerland	1	8	0.5	14.3	5.7	2.5	3.4	3.3	1.0
Belgium	1	7	0.5	0.0	1.1	0.0	1.1	1.3	0.9
Finland	1	6	0.4	16.7	0.9	17.9	0.2	0.6	0.3
Countries awarding none of the 99 prizes (the 5 with the largest pool shares), and all others:									
China (mainland)	0	0	0.0	–	–	–	1.5	3.5	0.4
Australia	0	0	0.0	–	–	–	2.3	3.5	0.7
France	0	0	0.0	–	–	–	5.2	3.2	1.6
Russia	0	0	0.0	–	–	–	1.3	1.0	1.3
Austria	0	0	0.0	–	–	–	1.7	0.9	1.9
All other countries (99)	0	0	0.0	–	–	–	6.8	6.2	1.1

Note: This table counts prizes and 2015–2024 recognitions by the country of the awarding organization; the three international prizes (Fields Medal, ICTP Ramanujan Prize, IABSE Award of Merit) have no national home. Home shares cover the 1,399 recognitions of national prizes with a placeable laureate: actual is the share of laureates based in the awarding country by the award year; expected is the share of the field's benchmark pool (the 1,000 researchers with the most citations to small-team papers, per field and award year) based there. The last three columns cover all 1,424 placeable recognitions: the share received by laureates based in the country (actual) and the country's share of the benchmark pool, weighted by the recognitions' field-year mix (expected); a researcher based in two countries counts in both. Ratios divide actual by expected. In the home-bias columns of the Hong Kong and Taiwan rows, mainland China, Hong Kong and Taiwan count as one home country on both sides; the received-share columns keep them apart. The last block lists the 5 non-awarding countries with the largest pool shares; the remaining countries are pooled in one row.

Table S4. Contextual Boolean Terms for Ambiguous Prize Names (Media Cloud Queries)

Prize	Search term	Context term(s)
Benjamin Franklin Medal in Chemistry	Benjamin Franklin Medal	Chemistry
Benjamin Franklin Medal in Electrical Engineering	Benjamin Franklin Medal	Electrical Engineering
Benjamin Franklin Medal in Life Science	Benjamin Franklin Medal	Life Science
Benjamin Franklin Medal in Materials Science	Benjamin Franklin Medal	Materials Science
Benjamin Franklin Medal in Mechanical Engineering	Benjamin Franklin Medal	Mechanical Engineering
Bower Award and Prize for Achievement in Science	Bower Award	science
Brain Prize	Brain Prize	neuroscience OR Lundbeck
Bruce Medal	Bruce Medal	astronomy OR Astronomical Society

(Table S4 continued)

Prize	Search term	Context term(s)
Crafoord Prize in Polyarthritis	Crafoord Prize	polyarthritis OR rheumatology
Frontiers of Knowledge Award (Basic Sciences)	Frontiers of Knowledge Award	Basic Sciences
Frontiers of Knowledge Award (Biology & Biomedicine)	Frontiers of Knowledge Award	biology OR biomedicine
Frontiers of Knowledge Award (Climate Change)	Frontiers of Knowledge Award	climate change
Frontiers of Knowledge Award (Ecology & Conservation)	Frontiers of Knowledge Award	ecology; conservation
Frontiers of Knowledge Award (Economics, Finance & Management)	Frontiers of Knowledge Award	economics OR finance OR management
Gold Medal for Astronomy	Gold Medal	Royal Astronomical Society
Harvey Prize	Harvey Prize	Technion
Heineken Prize for Biochemistry and Biophysics	Heineken Prize	biochemistry OR biophysics
Heineken Prize for Environmental Sciences	Heineken Prize	environment
Heineken Prize for Medicine	Heineken Prize	medicine
International Award of Merit in Structural Engineering	Award of Merit	structural engineering
Isaac Newton Medal	Isaac Newton Medal	physics; Institute of Physics
James Craig Watson Medal	Watson Medal	astronomy
Rolf Schock Prize in Mathematics	Rolf Schock Prize / Schock Prize	mathematics
Russ Prize	Russ Prize	engineering OR National Academy
Tang Prize in Biopharmaceutical Science	Tang Prize	biopharmaceutical

Note: For prizes whose name could plausibly match unrelated entities in news text, we augment the exact-phrase search with Boolean AND terms: (*prize name*) AND (*context terms*). The table lists the 25 prizes treated this way, together with the short search term and the context terms used to disambiguate. Terms separated by a semicolon or a slash are run as separate expressions and combined with OR.

Table S5. Correlation Between Normalized Prestige Indicators

	Survey Rating	Daily Page Views	Prize Age	Money per Prize	News Mentions
Survey Rating	1				
Daily Page Views	0.68 (0.08)***	1			
Prize Age	0.08 (0.12)	0.17 (0.10)	1		
Money per Prize	0.21 (0.11)	0.09 (0.10)	-0.47 (0.09)***	1	
News Mentions	0.53 (0.10)***	0.74 (0.07)***	-0.02 (0.10)	0.33 (0.10)***	1

Note: The table shows estimates of the Pearson correlation coefficients between each pair of the five prestige indicators, after normalization. Standard errors in parentheses. * $p<0.05$, ** $p<0.01$, *** $p<0.001$.

Table S6. Most Prestigious Prizes, Ranked

Award Name	Tier	Rank	Rating	Field
Nobel Prize in Physics	1	1	100.0	Physics and Astronomy
Nobel Prize in Physiology or Medicine	1	2	97.2	Life and Health Sciences
Sveriges Riksbank Prize in Economic Sciences in Memory of Alfred Nobel	1	3	96.7	Business and Economics
Nobel Prize in Chemistry	1	4	96.2	Chemistry
Fields Medal	1	5	89.4	Mathematics
A. M. Turing Award	1	6	87.8	Computing, Electrical
Abel Prize	1	7	85.2	Mathematics
Tyler Prize for Environmental Achievement	1	8	68.2	Environment
Japan Prize	2	9	66.1	Multidisciplinary
Breakthrough Prize in Fundamental Physics	2	10	64.4	Physics and Astronomy
Wolf Prize in Mathematics	2	11	64.0	Mathematics
Wolf Prize in Physics	2	12	63.4	Physics and Astronomy
World Food Prize	2	13	63.1	Ag. and Natural Resources
Templeton Prize	2	14	61.8	Multidisciplinary
Wolf Prize in Chemistry	2	15	61.7	Chemistry
Albert Lasker Basic Medical Research Award	2	16	61.2	Life and Health Sciences
IEEE Medal of Honor	2	17	60.4	Computing, Electrical
Lasker–DeBakey Clinical Medical Research Award	2	17	60.4	Life and Health Sciences
Shaw Prize in Mathematical Sciences	2	17	60.4	Mathematics
Stockholm Water Prize	2	20	60.1	Environment
Canada Gairdner International Award	3	21	58.5	Life and Health Sciences
Shaw Prize in Astronomy	3	22	58.1	Physics and Astronomy
Millennium Technology Prize	3	23	57.9	Multidisciplinary
Eni Award	3	23	57.9	Environment
Breakthrough Prize in Life Sciences	3	25	57.7	Life and Health Sciences
Queen Elizabeth Prize for Engineering	3	26	57.2	Multidisciplinary
Kyoto Prize in Basic Sciences	3	27	56.8	Multidisciplinary
Copley Medal	3	28	56.5	Multidisciplinary
Blue Planet Prize	3	29	56.0	Environment
Breakthrough Prize in Mathematics	3	30	55.9	Mathematics
Balzan Prizes	3	31	55.3	Multidisciplinary
Shaw Prize in Life Science and Medicine	3	32	54.7	Life and Health Sciences
Wolf Prize in Medicine	3	33	54.5	Life and Health Sciences
Charles Stark Draper Prize for Engineering	3	34	54.3	Multidisciplinary
Kavli Prize in Astrophysics	3	35	53.8	Physics and Astronomy
Crafoord Prize in Geosciences	3	36	52.2	Earth Sciences
Kyoto Prize in Advanced Technology	3	37	52.0	Multidisciplinary
Volvo Environment Prize	3	38	51.1	Environment
Crafoord Prize in Astronomy	3	39	50.7	Physics and Astronomy
Kavli Prize in Neuroscience	3	40	50.4	Life and Health Sciences
Kavli Prize in Nanoscience	3	40	50.4	Multidisciplinary
Crafoord Prize in Mathematics	3	42	49.9	Mathematics
Albert Einstein World Award of Science	3	43	49.6	Multidisciplinary
Canada Gairdner Global Health Award	3	44	49.4	Life and Health Sciences
King Faisal International Prize in Science	3	45	49.2	Multidisciplinary
Vetlesen Prize	3	46	49.1	Earth Sciences
Crafoord Prize in Biosciences	3	46	49.1	Life and Health Sciences
Robert Koch Award	3	48	48.0	Life and Health Sciences
Gold Medal for Astronomy	3	49	47.6	Physics and Astronomy

(Table S6 continued)

Award Name	Tier	Rank	Rating	Field
Marconi Prize	3	50	47.0	Computing, Electrical
Gödel Prize	3	50	47.0	Computing, Electrical
ICTP Ramanujan Prize	3	52	46.9	Mathematics
Frontiers of Knowledge Award in Climate Change	3	53	45.8	Earth Sciences
Von Hippel Award	3	54	45.3	Materials and Mining Eng.
Frontiers of Knowledge Award in Ecology and Conservation Biology	3	55	45.1	Environment
Timoshenko Medal	3	56	44.0	Multidisciplinary
Paul Ehrlich and Ludwig Darmstaedter Prize	3	57	43.7	Life and Health Sciences
Erwin Plein Nemmers Prize in Economics	3	58	43.5	Business and Economics
Stockholm Prize in Criminology	3	58	43.5	Other Social Sciences
Frontiers of Knowledge Award in Economics, Finance and Management	3	60	42.8	Business and Economics
Brain Prize	3	60	42.8	Life and Health Sciences
Wolf Prize in Agriculture	3	62	42.4	Ag. and Natural Resources
Tang Prize in Biopharmaceutical Science	3	62	42.4	Life and Health Sciences
Benjamin Franklin Medal in Electrical Engineering	3	64	41.8	Computing, Electrical
Rolf Schock Prize in Mathematics	3	65	41.6	Mathematics
Harvey Prize	3	66	41.5	Multidisciplinary
Benjamin Franklin Medal in Chemistry	3	67	40.8	Chemistry
Isaac Newton Medal	3	67	40.8	Physics and Astronomy
Benjamin Franklin Medal in Life Science	3	69	40.0	Life and Health Sciences
Gruber Prize in Cosmology	3	70	38.9	Physics and Astronomy
James Craig Watson Medal	3	71	38.1	Physics and Astronomy
Louisa Gross Horwitz Prize	3	72	37.7	Life and Health Sciences
Bower Award and Prize for Achievement in Science	3	73	37.6	Multidisciplinary
Maryam Mirzakhani Prize in Mathematics	3	74	37.5	Mathematics
Crafoord prize in Polyarthritis	3	75	37.0	Life and Health Sciences
Heineken Prize for Environmental Sciences	3	76	36.8	Environment
Warren Alpert Foundation Prize	3	77	35.7	Life and Health Sciences
Max Planck-Humboldt Research Award	3	78	35.6	Multidisciplinary
Faraday Lectureship Prize	3	79	35.5	Chemistry
Benjamin Franklin Medal in Mechanical Engineering	3	79	35.5	Mechanical Eng.
Stein Rokkan Prize for Comparative Social Science Research	3	81	35.1	Business and Economics
Max Planck Medal	3	82	34.2	Physics and Astronomy
Bruce Medal	3	83	33.9	Physics and Astronomy
Lorentz Medal	3	84	33.1	Physics and Astronomy
Heineken Prize for Medicine	3	84	33.1	Life and Health Sciences
InBev-Baillet Latour Health Prize	3	86	33.0	Life and Health Sciences
Russ Prize	3	87	32.8	Bio. and Biomedical Eng.
Wollaston Medal	3	88	32.6	Earth Sciences
Arthur L. Day Prize and Lectureship	3	89	32.3	Earth Sciences
Frontiers of Knowledge Award in Biology and Biomedicine	3	90	32.1	Life and Health Sciences
Benjamin Franklin Medal in Materials Science and Engineering	3	91	31.9	Materials and Mining Eng.

(Table S6 continued)

Award Name	Tier	Rank	Rating	Field
Gruber Prize in Genetics	3	92	31.8	Life and Health Sciences
Max Planck Research Award	3	92	31.8	Multidisciplinary
Heineken Prize for Biochemistry and Biophysics	3	94	31.5	Life and Health Sciences
Keio Medical Science Prize	3	95	30.3	Life and Health Sciences
International Award of Merit in Structural Engineering	3	96	29.6	Civil, Env., and Transp. Eng.
Matteucci Medal	3	97	29.2	Physics and Astronomy
Frontiers of Knowledge Award in Basic Sciences	3	98	28.7	Multidisciplinary
Gruber Prize in Neuroscience	3	99	27.6	Life and Health Sciences

Note: This table lists the 99 most prestigious prizes, ranked by Rating: the sum of the prestige indicators weighted by their first-principal-component loadings, rescaled so that the Nobel Prize in Physics equals 100. Prizes with the same Rating to one decimal share a Rank. Tiers follow the cumulative share of yearly recognition events (one person recognized with one prize): Tier 1 holds the top 10%, Tier 2 the next 20%, Tier 3 the rest.

Table S7. Awards by Field

Field	Prizes	Recognitions/year	Avg. Rank
Mathematics and Physical Sciences	27	46.8	42.3
Mathematics	9	11.8	33.9
Physics and Astronomy	14	28.9	51.6
Chemistry	4	6.0	41.2
Applied Sciences	17	19.8	67.6
Earth Sciences	5	3.8	62.4
Ag. and Natural Resources	2	4.0	37.5
Materials and Mining Eng.	2	2.0	72.5
Bio and Biomedical Eng.	1	1.5	87.0
Computing, Electrical	5	6.5	37.6
Civil, Env., and Transp. Eng.	1	1.0	96.0
Mechanical Eng.	1	1.0	80.0
Engineering, other	–	–	–
Life and Health Sciences	25	49.8	57.6
Psychology	–	–	–
Social Sciences	5	6.0	54.8
Business and Economics	4	5.0	50.5
Other Social Sciences	1	1.0	59.0
Environment and Multidisciplinary	25	39.2	40.7
Total	99	161.7	50.0

Note: This table summarizes the number of prizes, recognition events per year, and average rank (PCA-based) for the 99 most prestigious prizes, grouped by field. A dash marks a field with no prize.

Table S8. Robustness Exercise – Distribution of Prize Ranking under Random Weighting

Award Name	PCA Rank	P5	P50	P95	PCA Tier	% in right Tier
Nobel Prize in Physics	1	1	1	1	1	100.0
Nobel Prize in Physiology or Medicine	2	2	2	3	1	100.0
Sveriges Riksbank Prize in Economic Sciences in Memory of Alfred Nobel	3	3	4	6	1	98.9
Nobel Prize in Chemistry	4	3	3	4	1	100.0
Fields Medal	5	4	5	9	1	95.9
A. M. Turing Award	6	5	6	7	1	98.1
Abel Prize	7	5	7	27	1	80.9
Tyler Prize for Environmental Achievement	8	8	11	23	1	27.9
Japan Prize	9	9	13	28	2	94.4
Breakthrough Prize in Fundamental Physics	10	9	21	70	2	47.0
Wolf Prize in Mathematics	11	8	16	41	2	47.2
Wolf Prize in Physics	12	10	18	32	2	73.6
World Food Prize	13	7	20	65	2	38.7
Templeton Prize	14	8	15	55	2	42.2
Wolf Prize in Chemistry	15	12	21	35	2	58.5
Albert Lasker Basic Medical Research Award	16	11	16	27	2	75.9
IEEE Medal of Honor	17	7	11	25	2	54.6
Lasker–DeBakey Clinical Medical Research Award	18	11	16	25	2	84.2
Shaw Prize in Mathematical Sciences	19	10	25	58	2	41.0
Stockholm Water Prize	20	13	25	49	2	44.4
Canada Gairdner International Award	21	14	20	28	3	36.6
Shaw Prize in Astronomy	22	16	29	61	3	68.7
Millennium Technology Prize	23	17	30	62	3	88.9
Eni Award	24	14	35	70	3	85.0
Breakthrough Prize in Life Sciences	25	13	38	83	3	79.9
Queen Elizabeth Prize for Engineering	26	20	39	81	3	97.5
Kyoto Prize in Basic Sciences	27	17	26	44	3	58.4
Copley Medal	28	4	15	48	3	36.0
Blue Planet Prize	29	10	35	68	3	69.2
Breakthrough Prize in Mathematics	30	13	45	90	3	88.3
Balzan Prizes	31	13	24	42	3	58.3
Shaw Prize in Life Science and Medicine	32	25	35	65	3	96.8
Wolf Prize in Medicine	33	26	33	43	3	97.2
Charles Stark Draper Prize for Engineering	34	28	33	47	3	99.2
Kavli Prize in Astrophysics	35	19	42	75	3	83.6
Crafoord Prize in Geosciences	36	9	34	74	3	63.3
Kyoto Prize in Advanced Technology	37	23	36	56	3	84.3
Volvo Environment Prize	38	21	43	68	3	84.0
Crafoord Prize in Astronomy	39	14	38	69	3	71.2
Kavli Prize in Neuroscience	40	35	48	76	3	100.0
Kavli Prize in Nanoscience	41	30	49	78	3	97.8
Crafoord Prize in Mathematics	42	13	40	76	3	71.3
Albert Einstein World Award of Science	43	35	50	75	3	100.0
Canada Gairdner Global Health Award	44	36	58	85	3	100.0
King Faisal International Prize in Science	45	39	45	52	3	100.0
Vetlesen Prize	46	25	37	53	3	77.9
Crafoord Prize in Biosciences	47	30	41	53	3	98.3
Robert Koch Award	48	36	44	53	3	99.9
Gold Medal for Astronomy	49	8	40	92	3	69.1
Marconi Prize	50	26	50	80	3	98.2
Gödel Prize	51	26	65	88	3	98.3
ICTP Ramanujan Prize	52	34	71	89	3	100.0
Frontiers of Knowledge Award in Climate Change	53	41	61	87	3	100.0

(Table S8 continued)

Award Name	PCA Rank	P5	P50	P95	PCA Tier	% in right Tier
Von Hippel Award	54	29	57	83	3	92.3
Frontiers of Knowledge Award in Ecology and Conservation Biology	55	38	64	89	3	100.0
Timoshenko Medal	56	34	56	87	3	98.2
Paul Ehrlich and Ludwig Darmstaedter Prize	57	31	51	64	3	96.5
Erwin Plein Nemmers Prize in Economics	58	49	60	72	3	100.0
Stockholm Prize in Criminology	59	55	70	85	3	100.0
Frontiers of Knowledge Award in Economics, Finance and Management	60	48	70	88	3	100.0
Brain Prize	61	35	72	94	3	99.9
Wolf Prize in Agriculture	62	40	61	82	3	100.0
Tang Prize in Biopharmaceutical Science	63	31	77	96	3	99.9
Benjamin Franklin Medal in Electrical Engineering	64	10	47	78	3	72.4
Rolf Schock Prize in Mathematics	65	56	68	79	3	100.0
Harvey Prize	66	46	60	73	3	100.0
Benjamin Franklin Medal in Chemistry	67	11	52	79	3	78.2
Isaac Newton Medal	68	53	85	96	3	100.0
Benjamin Franklin Medal in Life Science	69	12	54	81	3	80.4
Gruber Prize in Cosmology	70	53	73	87	3	100.0
James Craig Watson Medal	71	19	58	79	3	86.9
Louisa Gross Horwitz Prize	72	44	80	98	3	100.0
Bower Award and Prize for Achievement in Science	73	10	52	78	3	81.1
Maryam Mirzakhani Prize in Mathematics	74	66	90	99	3	100.0
Crafoord prize in Polyarthritis	75	56	78	89	3	100.0
Heineken Prize for Environmental Sciences	76	50	72	93	3	100.0
Warren Alpert Foundation Prize	77	49	73	93	3	100.0
Max Planck-Humboldt Research Award	78	52	88	99	3	100.0
Faraday Lectureship Prize	79	21	67	91	3	89.5
Benjamin Franklin Medal in Mechanical Engineering	80	15	64	88	3	84.9
Stein Rokkan Prize for Comparative Social Science Research	81	59	77	94	3	100.0
Max Planck Medal	82	31	85	98	3	96.7
Bruce Medal	83	28	80	97	3	94.0
Lorentz Medal	84	37	85	98	3	97.5
Heineken Prize for Medicine	85	66	80	94	3	100.0
InBev-Baillet Latour Health Prize	86	55	78	92	3	100.0
Russ Prize	87	67	86	94	3	100.0
Wollaston Medal	88	20	80	97	3	91.0
Arthur L. Day Prize and Lectureship	89	47	80	97	3	100.0
Frontiers of Knowledge Award in Biology and Biomedicine	90	62	90	98	3	100.0
Benjamin Franklin Medal in Materials Science and Engineering	91	17	74	92	3	88.6
Gruber Prize in Genetics	92	68	87	95	3	100.0
Max Planck Research Award	93	44	78	98	3	99.3
Heineken Prize for Biochemistry and Biophysics	94	51	78	94	3	99.9
Keio Medical Science Prize	95	74	88	97	3	100.0
International Award of Merit in Structural Engineering	96	64	94	99	3	100.0
Matteucci Medal	97	25	90	99	3	93.7
Frontiers of Knowledge Award in Basic Sciences	98	74	94	99	3	100.0

(Table S8 continued)

Award Name	PCA Rank	P5	P50	P95	PCA Tier	% in right Tier
Gruber Prize in Neuroscience	99	73	93	99	3	100.0

Note: This table shows the rank and tier of each of the 99 prizes across 1,000 rankings built with random weights over the five standardized indicators. PCA Rank and PCA Tier are the original ranking and tier; P5, P50 and P95 the 5th, 50th and 95th percentiles of the simulated rank; % in right Tier the share of simulations that place the prize in its original tier.

Table S9. Selected Early Career Prizes, Ranked

Award Name	Tier	Field
Bôcher Memorial Prize	1	Mathematics
Clay Research Award	1	Mathematics
COPSS Presidents' Award	1	Mathematics
Fermat Prize	1	Mathematics
Fields Medal	1	Mathematics
ICTP Ramanujan Prize	1	Mathematics
Max Planck-Humboldt Research Award	1	Multidisciplinary
Oswald Veblen Prize in Geometry	1	Mathematics
Rollo Davidson Prize	1	Mathematics
Salem Prize	1	Mathematics
Annie Jump Cannon Award in Astronomy	2	Astronomy
Berwick Prize	2	Mathematics
Bigsby Medal	2	Earth Sciences
Fernand Holweck Medal and Prize	2	Physics
ICTP Prize	2	Physics
IJCAI Computers and Thought Award	2	Computer Science and Engineering
IMU Abacus Medal	2	Computer Science and Engineering
J. H. Wilkinson Prize for Numerical Software	2	Computer Science and Engineering
Leslie Fox Prize for Numerical Analysis	2	Mathematics
Lieben Prize	2	Multidisciplinary
Loève Prize	2	Mathematics
Machtey Award	2	Computer Science and Engineering
Michael Brin Prize in Dynamical Systems	2	Mathematics
Overton Prize	2	Life Sciences and Medicine
Peccot Lectures	2	Mathematics
Ruth Lyttle Satter Prize in Mathematics	2	Mathematics
Sloan Research Fellowship∗	2	Multidisciplinary
Troland Research Awards	2	Psychology
Walter L. Huber Civil Engineering Research Prize	2	Computer Science and Engineering
Yrjö Jahnsson Award	2	Social Sciences
Adolph Lomb Medal	3	Physics
Amelia Earhart Fellowship∗	3	Computer Science and Engineering
Beilby Medal and Prize	3	Chemistry
Bunsen–Kirchhoff Award	3	Chemistry
Colworth Medal	3	Life Sciences and Medicine
Dahl–Nygaard Prize Junior Prize	3	Computer Science and Engineering
E. H. Moore Research Article Prize	3	Mathematics
E. W. Beth Dissertation Prize	3	Multidisciplinary
EMBO Gold Medal	3	Life Sciences and Medicine
European Prize in Combinatorics	3	Mathematics
Fröhlich Prize	3	Mathematics
Germán Bernácer Prize	3	Social Sciences
Gossen Prize	3	Social Sciences
Gribov Medal	3	Physics
Hausdorff Medal	3	Mathematics
James B. Macelwane Medal	3	Earth Sciences

(Table S9 continued)

Award Name	Tier	Field
James Clerk Maxwell Medal and Prize	3	Physics
Kalai Prize	3	Computer Science and Engineering
Levi L. Conant Prize	3	Mathematics
LTPP Data Analysis Contest - Challenge Category	3	Computer Science and Engineering
Luigi G. Napolitano Award	3	Computer Science and Engineering
Margaret Mead Award	3	Social Sciences
Maurice Wilkes Award	3	Computer Science and Engineering
Nerode Prize	3	Mathematics
New Horizons in Fundamental Physics	3	Physics
New Horizons in Mathematics	3	Mathematics
Otto Hahn Medal – Biological-Medical	3	Life Sciences and Medicine
Otto Hahn Medal – Chemical-Physical-Engineering	3	Multidisciplinary
Otto Hahn Medal – Social Sciences-Humanities	3	Multidisciplinary
Presburger Award	3	Computer Science and Engineering
Ribenboim Prize	3	Mathematics
Richard Lounsbery Award	3	Life Sciences and Medicine
Science Innovation Award	3	Earth Sciences
Stampacchia Medal	3	Mathematics
Stockholm Junior Water Prize	3	Multidisciplinary
T. S. Ashton Prize	3	Social Sciences
W. Alden Spencer Award	3	Life Sciences and Medicine
Wayne B. Nottingham Prize	3	Physics

Note: This table lists the 68 most prestigious early-career prizes, selected and tiered as described in the section "Early career prize methodology": Tier 1 is the top 10 prizes, Tier 2 the next 20. Within tiers, prizes are alphabetical. $*$ Research fellowship: a cohort program funding a period of research rather than a single award.

Table S10. Prizes with the Largest Home-Bias Excess Against the Field Benchmark

Prize	Country	N	Home	Bench.	Excess
Max Planck Medal	Germany	10	0.90	0.13	+0.77
Copley Medal	United Kingdom	9	0.78	0.12	+0.66
Gold Medal of the Royal Astronomical Society	United Kingdom	20	0.75	0.11	+0.64
Matteucci Medal	Italy	8	0.62	0.03	+0.59
Lorentz Medal	Netherlands	2	0.50	0.03	+0.47
Keio Medical Science Prize	Japan	20	0.50	0.04	+0.46
Benjamin Franklin Medal in Mechanical Engineering	United States	4	1.00	0.54	+0.46
Max Planck Research Award	Germany	4	0.50	0.04	+0.46
Benjamin Franklin Medal in Electrical Engineering	United States	9	1.00	0.54	+0.46
Benjamin Franklin Medal in Materials Science and Engineering	United States	2	1.00	0.57	+0.43
Largest negative excess:					
Godel Prize	United States	26	0.62	0.73	−0.12
Breakthrough Prize in Mathematics	United States	14	0.64	0.69	−0.05
Vetlesen Prize	United States	5	0.60	0.64	−0.04
Heineken Prize for Environmental Sciences	Netherlands	4	0.00	0.04	−0.04

Note: This table lists the prizes whose laureates are most often based in the awarding country relative to the field benchmark: the field's 1,000 researchers with the most citations to small-team papers, located by their affiliations up to the award year. N is the number of 2015–2024 recognitions; the smallest N are noisy. Home and Bench. are the shares of the prize's laureates and of the benchmark researchers based in the awarding country; Excess is their difference.

Early career prize methodology

For early career prizes, we make the inclusion criteria more lenient to capture more prizes. We rely more on prize age and online popularity, since these are more readily available for such awards. We define eligible prizes as those with any age or career stage restrictions or those focusing explicitly on recent work (past 10 years). Selection requires meeting one of:

- Two or more daily Wikipedia page views
- At least 60 years of history
- Inclusion in Wikipedia's early career awards list

We maintain the exclusion criteria regarding international reach and selection procedures, except that we do not require winner lists to be readily available. This strategy identifies the 68 most prestigious international early career prizes, which produce about 265 recognition events each year. We provide a tier-based ranking for these prizes, based on a prestige proxy that puts 80% weight on daily page views and 20% weight on prize age (both normalized). We assign the first ten prizes to the first tier and the next twenty to the second tier.

References

1. Marx M, Fuegi A. Reliance on Science: Worldwide Front-Page Patent Citations to Scientific Articles. Strategic Management Journal. 2020;41(9):1572-94. doi:10.1002/smj.3145.
2. Marx M, Fuegi A. Reliance on Science by Inventors: Hybrid Extraction of In-Text Patent-to-Article Citations. Journal of Economics & Management Strategy. 2022;31(2):369-92. doi:10.1111/jems.12455.
3. Marx M. Reliance on Science. Zenodo; 2026. Data set, patent-to-paper citations file pcs_oa_uspto.csv, published 22 July 2026. doi:10.5281/zenodo.21493744.
